# Climate-driven habitat shifts reveal contrasting climate association of four *Stipa* species in Central Asia

**DOI:** 10.3389/fpls.2026.1815614

**Published:** 2026-06-24

**Authors:** Aogan, Alatanzhula, Yi-Ding Yin, Li-Qing Zhao

**Affiliations:** 1 Ministry of Education Key Laboratory of Ecology and Resource Use of the Mongolian Plateau and Inner Mongolia Key Laboratory of Grassland Ecology, School of Ecology and Environment, Inner Mongolia University, Hohhot, China; 2 Inner Mongolia Research Academy of Eco-Environmental Sciences, Hohhot, China

**Keywords:** Biomod2, Central Asia, climate change, potential geographic distribution, *Stipa* L.

## Abstract

Climate change is reshaping global plant biogeography, and understanding the distribution dynamics of dominant species is critical for sustainable ecosystem management. *Stipa* species dominate the grasslands of Central Asia, constitute a major component of regional steppe ecosystems, and provide essential forage resources for livestock production. Four constructive *Stipa* species sensitive to temperature and precipitation changes (*S. breviflora*, *S. bungeana*, *S. grandis*, and *S. klemenzii*) were selected for analysis. Current and future habitat suitability and distribution trends were predicted using the ensemble modeling framework in Biomod2 (ensemble AUC/TSS > 0.9; individual model selection based on TSS > 0.8). Under future climate scenarios, the suitable habitat centroids of all four species were projected to shift westward. The warm- and dry-adapted *S. breviflora* exhibited a relatively stable distribution range, with its changes being primarily driven by elevation. In contrast, the warm- and humid-adapted *S. bungeana* showed a continuous northwestward expansion, mainly influenced by the mean temperature of the coldest quarter. Conversely, the cold- and humid-adapted *S. grandis* and cold- and dry-adapted *S. klemenzii*, were predicted to experience significant habitat contraction under future climate scenarios. The distributions of both species were primarily influenced by precipitation during the coldest quarter. Overall, cold-adapted species such as *S. grandis* and *S. klemenzii* are expected to be more vulnerable to future climate change, despite a gradual slowdown in the rate of suitable habitat contraction over time. Therefore, grassland conservation and management in Central Asia should dynamically adjust conservation priorities, with greater attention given to the contraction and migration trends of these cold-adapted species in order to enhance ecosystem stability and adaptive capacity.

## 1 Introduction

The arid and semi-arid grasslands of Central Asia constitute an important component of the Eurasian Steppe and represent one of the largest temperate grassland ecosystems in the world. These grasslands provide critical ecological and economic functions, including maintaining biodiversity, preventing soil erosion, regulating regional climate, and supporting extensive pastoral livelihoods (Xie et al., 2003; Kang et al., 2007; Gao et al., 2018; Qiqige et al., 2023). However, the region is highly sensitive to global climate change and anthropogenic disturbances, particularly increasing temperatures, altered precipitation regimes, overgrazing, and land-use change, all of which have accelerated grassland degradation and desertification in recent decades (Hilker et al., 2014; Huang et al., 2016; Gao et al., 2018; Yan et al., 2024). Such environmental pressures have greatly threatened the stability and resilience of grassland ecosystems, making the arid and semi-arid regions of Central Asia among the most vulnerable ecosystems under future climate scenarios. Consequently, this region has become a priority area for biodiversity conservation and ecological restoration.

Climate change is one of the major drivers shaping the geographical distributions and ecological adaptability of plant species (Yang et al., 2023; Pérez-Suárez et al., 2024; Hu et al., 2025), especially in arid and semi-arid ecosystems where vegetation is highly sensitive to environmental fluctuations. Recent climatic changes are expected to substantially alter habitat suitability, population dynamics, and species interactions in grassland ecosystems (Jennings and Harris, 2017; Ofori et al., 2017; Auber et al., 2022; Archibald et al., 2024; Bhuyan et al., 2025). As dominant components of the Central Asia Steppe, grassland plant species play crucial roles in maintaining ecosystem functioning, productivity, and resilience. Therefore, understanding how characteristic grassland species respond to future climate change is essential for predicting biodiversity dynamics and developing effective conservation and sustainable management strategies in Central Asia. To achieve this, species distribution models (SDMs) provide an effective framework for linking species occurrences with environmental variables to predict potential distribution shifts under climate change (Sarkar et al., 2024; Soilhi et al., 2025), and are widely used in biogeographical and ecological studies.

Previous studies have shown that the distribution patterns of *Stipa* species are mainly controlled by temperature, precipitation, and their seasonality, while topographic factors such as elevation also play important roles, especially in plateau and mountainous regions (Zhao, 2010; Han et al., 2011; Hu et al., 2021). Under future climate change scenarios, different *Stipa* species exhibit markedly different responses, including habitat expansion, contraction, relative stability, and shifts toward higher latitudes or elevations, indicating substantial interspecific heterogeneity in climate sensitivity and ecological adaptation (Wen et al., 2008; Hu et al., 2015; Yang, 2016; Ma and Sun, 2018; Lv and Zhou, 2018; Wang et al., 2019; Tu, 2020; Ma et al., 2022). In addition, climate change may affect not only the extent of suitable habitats, but also the spatial structure, connectivity, and fragmentation of *Stipa* communities (Zhang et al., 2025). As dominant and constructive species in the arid and semi-arid grasslands of Central Asia, *Stipa* species play crucial roles in maintaining primary productivity, soil stability, forage supply, and ecosystem resilience. Therefore, declines or shifts in *Stipa* distributions under future climate change may substantially alter grassland community composition, reduce ecosystem stability, and accelerate grassland degradation and desertification processes across Central Asian grasslands.

The genus *Stipa* is one of the major constructive taxa in the grasslands of Central Asia and plays an important role in maintaining regional ecological stability, ecosystem functioning, and carbon cycling (Hou et al., 2013). Influenced jointly by longitudinal, latitudinal, and elevational gradients, different *Stipa* species have formed distinct ecological differentiation patterns across Central Asia (Lu and Wu, 1996). For example, *Stipa breviflora* is primarily a warm- and dry-adapted species and is widely distributed in the warm-arid regions of the northwestern Loess Plateau, the southern Mongolian Plateau, and the Tibetan Plateau and Xinjiang (Lu and Wu, 1996; Zhao, 2010; Han et al., 2011). *S. bungeana* is a warm- and humid-adapted species and is mainly distributed in the relatively warm and humid areas south of the Yinshan Mountains, southeast of the Greater Khingan Mountains, and in the Tibetan Plateau and Xinjiang (Lu and Wu, 1996; Zhao, 2010; Han et al., 2011). *S. grandis* is a cold- and humid-adapted species and is primarily distributed in the cool and humid regions of the northeastern Mongolian Plateau (Lu and Wu, 1996; Zhao, 2010; Han et al., 2011). In contrast, *S. klemenzii* is a cold- and dry-adapted species and is mainly distributed in the cold-arid regions of the north-central Mongolian Plateau, the Tibetan Plateau, and Xinjiang (Lu and Wu, 1996; Zhao, 2010; Han et al., 2011). These contrasting adaptations to hydrothermal conditions suggest that *Stipa* species may differ substantially in their sensitivity and responses to climate change.

Therefore, against the broader background of the Eurasian continent, this study focuses on the core region of Central Asia and applies a multi-algorithm ensemble modeling approach to clarify the spatiotemporal dynamics of *Stipa* species with different ecological adaptations under climate change. We selected four representative *Stipa* species, *S. breviflora* Griseb., *S. bungeana* Trin., *S. grandis* P. A. Smirn., and *S. klemenzii* Roshev., and used the Biomod2 integrated model to predict their current and future suitable habitats and distribution trends. Specifically, this study addresses the following questions: (1) Do these four representative *Stipa* species differ significantly in their dependence on key environmental variables, including climate, topography, and soil? (2) How will their suitable habitats vary across space and time under different future climate scenarios, and how will their distribution centroids shift? (3) Do they show heterogeneous responses to climate change, and what mechanisms may underlie these differences?

## 2 Materials and methods

### 2.1 Species distribution data

This study focused on four *Stipa* species, including *S. breviflora*, *S. bungeana*, *S. grandis*, and *S. klemenzii*. A total of 84, 22, 47, and 38 occurrence records for these species, respectively, were obtained through field collection and species identification. These field-collected records served as first-hand occurrence data and provided reliable taxonomic verification for the study. To improve spatial coverage, additional occurrence records were compiled from multiple supplementary sources, including the Global Biodiversity Information Facility (GBIF, http://www.gbif.org/, accessed May 2025), the Chinese Virtual Herbarium (CVH, https://www.cvh.ac.cn/, accessed April-May 2025), the Herbarium of the Institute of Botany, Chinese Academy of Sciences (http://pe.ibcas.ac.cn, accessed May 2025), published literature (Nobis, 2014; Zhu, 2020), and the *Flora of Siberia* (Malyschev and Peschkova, 2001). In total, 1,090 raw occurrence records, including both field-collected and supplementary records, were obtained for the four species (**Supplementary Table 1**).

**Figure 1:**
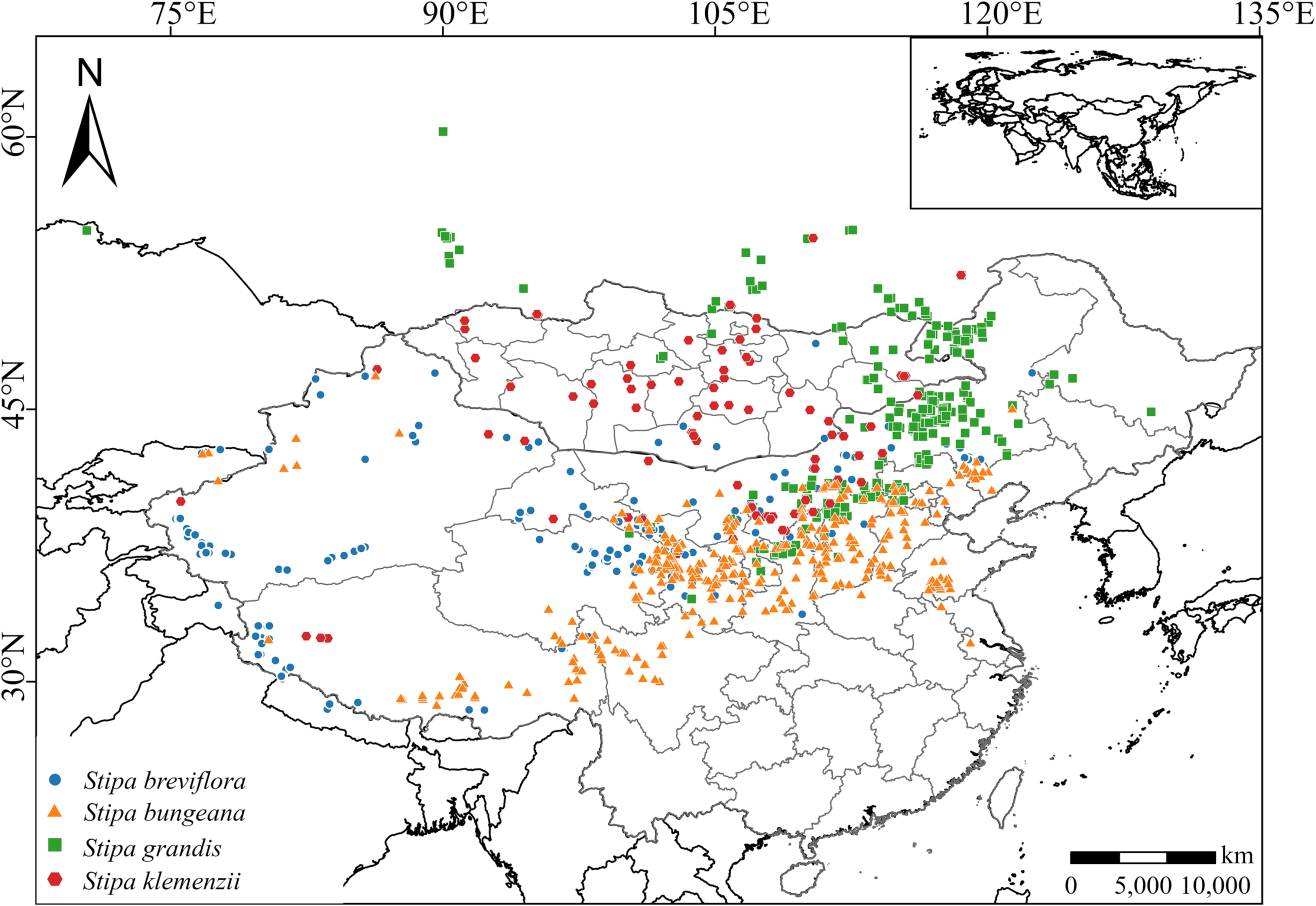
Geographic distribution occurrence records of four *Stipa* species. A total of 224 records were obtained for *S. breviflora*, 324 for *S. bungeana*, 253 for *S. grandis*, and 84 for *S. klemenzii*.

A standardized protocol was applied for data cleaning and filtering. Missing coordinates were retrieved using Google Earth Pro, and incomplete, duplicate, and potentially misidentified records were removed. Where possible, specimen images and associated taxonomic information were manually examined to exclude records showing obvious phenotypic inconsistencies or insufficient taxonomic reliability. To minimize spatial autocorrelation and reduce model overfitting, spatial thinning was performed using the spThin function (Aiello-Lammens et al., 2015) in R with a 10 × 10 km grid, retaining only one occurrence point per grid cell (Yang et al., 2023). This procedure was consistently applied across all species. After filtering and thinning, 885 valid occurrence records remained for subsequent modeling analyses, including 224 records for *S. breviflora*, 324 for *S. bungeana*, 253 for *S. grandis*, and 84 for *S. klemenzii* (**Figure 1**).

### 2.2 Environmental variables

Environmental predictors were categorized into climate, topography, and soil variables. Climate and topographic data were obtained from the WorldClim database (http://worldclim.org/, accessed July 2025) at 5 arc-minutes resolution (~ 9 km), including 19 bioclimatic variables (1970–2000 averages), solar radiation, wind speed, vapor pressure, and elevation. Slope and aspect were derived from elevation using ArcGIS 10.8. A total of 21 soil variables at 0–20 cm depth were extracted from the Harmonized World Soil Database (HWSD v2.0, http://www.fao.org/, accessed July 2025) (Liu et al., 2025). To ensure consistency, all variables were resampled to a uniform spatial resolution of 5 arc-minutes.

Future climate projections were obtained from the WorldClim database (accessed July 2025) using the BCC-CSM2-MR model (CMIP6 framework), which provides reliable simulations for the Eurasian continent. The BCC-CSM2-MR model is one of the most commonly used models for simulating the global climate response to increased greenhouse gas concentrations (Wu et al., 2019). Projections were generated for two future periods, including 2041-2060 (2050s) and 2061-2080 (2070s), under three Shared Socioeconomic Pathways (SSP126, SSP370, and SSP585) (**Supplementary Table 2**) (Eyring et al., 2016; Zhang et al., 2019). Under future climate scenarios, non-climatic variables such as soil and topographic factors were assumed to remain constant because topographic conditions are relatively stable over short evolutionary timescales, and globally consistent future projections of soil variables under SSP scenarios at comparable spatial resolutions are currently limited or unavailable. Therefore, maintaining these variables unchanged is a common practice in species distribution modeling to ensure temporal comparability among present and future projections (Zhao et al., 2025).

To minimize multicollinearity and improve model robustness (Xian et al., 2023; Lv et al., 2025), Pearson correlation analysis was first conducted in R (v4.5.0) on 46 candidate environmental variables (**Supplementary Table 3**) (Zhang et al., 2025). Variables having Pearson correlation coefficients |r| > 0.8 were removed to avoid multicollinearity among the independent variables, following commonly used practices in species distribution modeling (Dormann et al., 2013; Huang et al., 2023; Xu et al., 2023). For highly correlated variable pairs, preliminary MaxEnt simulations were subsequently performed in the MaxEnt software using default settings (e.g., 25% test percentage and 10-fold bootstrap replicates) to compare the percent contribution and Jackknife test scores of each variable (Elith et al., 2011). Only the variable with stronger explanatory power for species distribution was retained. In addition, variables with contribution rates lower than 1% were excluded from the final models. Consequently, the final variable sets consisted of 14, 12, 7, and 11 variables for the four *Stipa* species, respectively (**Supplementary Table 3**). Correlation matrices of the final selected variables for each species are provided in the **Supplementary Materials** (**Supplementary Figure 1**).

### 2.3 Ensemble modeling framework, evaluation metrics and model selection

Species distribution modeling was performed using the Biomod2 v4.2.6 package in R v4.5.0. Twelve algorithms were implemented, including artificial neural network (ANN), classification tree analysis (CTA), flexible discriminant analysis (FDA), generalized additive model (GAM), generalized boosting model (GBM), generalized linear model (GLM), multivariate adaptive regression splines (MARS), maximum entropy (MAXENT), random forests (RF), random forest downsampled (RFd), surface range envelope (SRE), and eXtreme Gradient Boosting Training (XGBOOST). For each species, 75% of occurrence records were randomly selected for model training and the remaining 25% were used for testing (Wang et al., 2019; Liu et al., 2024). The modeling procedure was repeated five times to reduce uncertainty associated with random data partitioning. Default parameter settings implemented in Biomod2 were used for all algorithms to maintain methodological consistency and comparability across species and modeling techniques. Although several machine-learning algorithms, such as RF, GBM, XGBOOST, and MAXENT, can be sensitive to parameter tuning, the use of unified default settings is a common practice in large ensemble species distribution modeling studies involving multiple algorithms and repeated simulations. In this study, our primary objective was to evaluate consensus distribution patterns across ensemble models rather than optimize the predictive performance of individual algorithms. Furthermore, repeated model runs and ensemble forecasting were expected to reduce uncertainties associated with individual model parameterization and improve prediction robustness.

Because true absence data were unavailable, pseudo-absence points were generated as alternative negative samples to distinguish species occurrences from background environmental conditions (Elith et al., 2006). For each species, 1,000 pseudo-absence points were independently generated within the study area using the Surface Range Envelope (SRE) strategy implemented in Biomod2 (Barbet-Massin et al., 2012). The SRE approach selects pseudo-absences outside environmentally suitable conditions defined by occurrence records, thereby reducing the likelihood of assigning pseudo-absences near known presence locations. To reduce uncertainty associated with random pseudo-absence selection, the pseudo-absence generation procedure was repeated three times (Xian et al., 2023). Although occurrence sample sizes differed among species, a uniform number of pseudo-absence points was adopted to maintain methodological consistency and comparability across species and algorithms. Previous studies have demonstrated that this pseudo-absence sample size provides reliable model performance while maintaining computational efficiency in ensemble species distribution modeling. All models were implemented using default parameter settings to maintain methodological consistency and comparability among algorithms (**Supplementary Table 4**). Combined with 12 modeling algorithms and five repeated runs, the modeling framework generated 180 candidate models per species, thereby improving prediction robustness. Model performance was evaluated using the True Skill Statistic (TSS) and the Area Under the Receiver Operating Characteristic Curve (AUC). While AUC assesses overall model discrimination ability across thresholds, TSS measures classification accuracy independent of species prevalence (Allouche et al., 2006). Models with mean TSS > 0.8 and AUC > 0.9 were retained for ensemble modeling. Ensemble models were used because they integrate predictions from multiple algorithms, thereby reducing model-specific uncertainty and improving predictive robustness and accuracy (Araújo and New, 2007). Three ensemble strategies, including Arithmetic Mean (EMmean), Weighted Mean (EMwmean), and Committee Averaging (EMca), were evaluated. In the EMwmean approach, model weights were assigned based on TSS values, while AUC was used as an additional evaluation criterion. Considering overall predictive performance and consistency across species, EMca was selected as the final ensemble strategy. The maximum TSS threshold was used to convert continuous model outputs into binary suitable and unsuitable habitat maps, and response curves were generated to characterize species-environment relationships (Lv et al., 2025).

### 2.4 Spatial analysis and mapping

ArcGIS Pro was used to project raster outputs to the Albers equal-area projection. Suitability values (0–1000, representing the scaled output of Biomod2 and equivalent to 0–1) were classified into four habitat suitability categories, unsuitable (0-25%), low suitability (25-50%), moderate suitability (50-75%), and high suitability (75-100%). This equal-interval classification approach was adopted to facilitate visual interpretation and comparison of spatial distribution patterns among species and climate scenarios. Although simple and widely used in species distribution studies, equal-interval classification may not fully capture species-specific ecological thresholds because the distribution of suitability values can vary among species and algorithms. Therefore, the resulting suitability classes should be interpreted as relative suitability gradients rather than absolute ecological thresholds. The area of each suitability class was quantified using raster spatial statistics based on cell counts and cell size.

### 2.5 Centroid calculation

To assess the changes in the spatial distribution of suitable habitats, binary distribution maps were reprojected using the Albers equal-area projection. By comparing the binary suitable habitat maps between the current and future periods, changes in habitat suitability were classified into three categories including, expansion (areas unsuitable under current conditions but suitable in the future), contraction (areas suitable under current conditions but unsuitable in the future), and stability (areas remaining suitable in both periods). Habitat gain and loss were calculated for the 2050s relative to the current and for the 2070s relative to the 2050s (Chen et al., 2025). Using the ArcGIS pro ‘Raster Calculator’ function to calculate the Mean Center, so as to determine the average centroid of the suitable area in each time period. Trajectory analysis was used to determine migration directions, and Euclidean distance was used to quantify migration magnitude. These analyses allowed characterization of long-term distributional responses of the four *Stipa* species under projected climate change.

## 3 Results

### 3.1 Model performance evaluation

The performance of the 12 individual models implemented within the Biomod2 framework was evaluated using TSS and AUC metrics. Models demonstrating strong predictive performance were selected for ensemble modeling. Specifically, models with TSS > 0.8 and AUC > 0.9 were considered suitable candidates for inclusion in the ensemble models, which generally showed improved or comparable predictive performance relative to most individual algorithms (**Supplementary Figure 2**; **Supplementary Table 5**).

For *S. breviflora*, an ensemble model was constructed using seven individual models, including CTA, FDA, GAM, GBM, GLM, MARS, and MAXENT (**Supplementary Figure 2a**). Among them, the MAXENT model showed the highest average AUC and TSS values (**Supplementary Table 5**). For *S. bungeana*, nine individual models were used to build the ensemble model, including CTA, GAM, GBM, GLM, MARS, MAXENT, RF, RFd, and XGBOOST (**Supplementary Figure 2b**), among which the MAXENT model exhibited the highest average AUC and TSS values (**Supplementary Table 5**). For *S. grandis*, an ensemble model was constructed using eight individual models, including CTA, GBM, GLM, MARS, MAXENT, RF, RFd, and XGBOOST (**Supplementary Figure 2c**). Among these, the GBM model showed the highest AUC value, while the MAXENT model had the highest average TSS value (**Supplementary Table 5**). For *S. klemenzii*, eight individual models were used to build the ensemble model, including CTA, FDA, GAM, GBM, GLM, MARS, MAXENT, and RFd (**Supplementary Figure 2d**). Among them, the RFd model achieved the highest average AUC value, while the MARS model produced the highest average TSS value (**Supplementary Table 5**).

Compared with the individual model, the AUC and TSS values of the ensemble model were higher than those of each individual model. The ensemble model constructed by the EM method significantly improved the prediction accuracy, with the AUC and TSS values of cross-validation results exceed 0.9, which proves that the integration of multiple algorithms enhances both prediction accuracy and robustness (**Supplementary Table 5**).

### 3.2 Effects of environmental variables on *Stipa* species

The contribution rates of environmental variables derived from the ensemble models (**Table 1**) revealed both shared and differentiated environmental controls across the four *Stipa* species. Elevation, temperature, and precipitation variables consistently exhibited the highest contributions, although their relative contribution rates differed among species (**Table 1**).

**Table 1:** Contribution of environmental variables to the suitable distribution of four *Stipa* species species (%).

*S. breviflora*		*S. bungeana*		*S. grandis*		*S. klemenzii*	
Environmental variable	Contribution	Environmental variable	Contribution	Environmental variable	Contribution	Environmental variable	Contribution
elev	25.51	bio11	38.16	bio19	54.71	bio19	27.46
bio9	24.14	bio19	34.11	bio18	26.44	bio9	21
srad	20.16	bio18	19.14	bio4	8.28	bio15	17.61
bio1	13.30	srad	3.69	wind	5.63	elev	12.28
ph_water	6.04	bio3	2.09	elev	3.91	bio18	11.63
bio19	3.16	bio15	0.98	D1_bsat	0.57	D1_alum_sat	3.88
D1_bulk	2.33	vapr	0.74	D1_alum_sat	0.46	bio4	1.94
bio11	1.65	slope	0.61			D1_bsat	1.78
slope	0.96	D1_bsat	0.12			D1_gypsum	1.29
D1_bsat	0.69	D1alum_sat	0.12			D1_coarse	0.81
bio18	0.69	D1_gypsum	0.12			aspect	0.32
bio2	0.69	D1_tcarbon_eq	0.12				
D1_gypsum	0.41						
D1_tcarbon_eq	0.27						

D1_bsat, base saturation of the topsoil layer; D1_alum_sat, aluminum saturation of the topsoil layer; D1_tcarbon_eq, total carbon content equivalent of the topsoil layer; D1_gypsum, gypsum content of the topsoil layer; D1_bulk, bulk density of the topsoil layer.

*Stipa breviflora* was primarily influenced by elevation (25.51%), the mean temperature of the driest quarter (bio9, 24.14%), and solar radiation (srad, 20.16%). These three environmental variables together contributed nearly 70% of the relative variable importance (**Table 1**). Among the environmental variables, elevation emerged as the most important driving factor. The response curve suggested that the species maintained relatively high predicted suitability across a broad elevational gradient, with suitability peaking between 2000 and 4000 m, indicating that this range may provide the most favorable environmental conditions. However, according to the *Flora of China*, *S. breviflora* is primarily distributed between 700 and 4700 m elevation (Wu et al., 2006). Therefore, the predicted suitability at extremely high elevations should be interpreted cautiously, as it may partly reflect model extrapolation beyond the environmental conditions represented in the occurrence records. For mean temperature of the driest quarter (bio9), high suitability was observed when its value ranges from -40 to 20°C, with a sharp decline in suitability above 20 °C. Additionally, suitability increased with higher solar radiation, remaining high when solar radiation exceeds 10,000 kJ m-2 day-1 (**Supplementary Figure 3**).

In contrast, the potential distribution of *S. bungeana* was mainly affected by the combined effects of mean temperature of the coldest quarter (bio11) and seasonal precipitation. The mean temperature of the coldest quarter had the highest contribution rate of 38.16%, followed by precipitation of the coldest quarter (bio19) at 34.11% (**Table 1**). Together, these two variables accounted for over 70% of the total contribution, with precipitation of the warmest season (bio18) providing additional explanatory power regarding seasonal water availability. The response curve showed that *S. bungeana* exhibits high suitability when the mean temperature of the coldest quarter (bio11) ranges from -18°Cand 18°C. For precipitation of the coldest quarter (bio19), *S. bungeana* showed the highest suitability under extremely low precipitation in the coldest season, and the suitability gradually decreased as precipitation increased. In contrast, habitat suitability remained stable with increasing precipitation of the warmest season (bio18) (**Supplementary Figure 4**).

In contrast to the other two species, *S. grandis* showed the strongest dependence on precipitation, with precipitation of the coldest quarter (bio19) contributing 54.71% and precipitation of the warmest season (bio18) contributing 26.44% (**Table 1**), indicating a high sensitivity to both the amount and seasonal distribution of precipitation. Response curve analysis showed that habitat suitability was highest when precipitation of the coldest quarter (bio19) ranged from 0 to 125 mm, and decreased when precipitation exceeded 125 mm. In contrast, habitat suitability was stable with increasing precipitation of the warmest season (bio18) (**Supplementary Figure 5**).

*Stipa klemenzii* displayed a relatively balanced influence from both precipitation and temperature. The precipitation of the coldest quarter (bio19) contributed 27.46%, mean temperature of the driest quarter (bio9) contributed 21%, and precipitation seasonality (bio15) contributed 17.61% (**Table 1**), indicating sensitivity to dry-season temperature and seasonal water availability. The response curve analysis showed that habitat suitability was high when precipitation of the coldest quarter (bio19) ranged from 0 to 250 mm, decreasing with increasing precipitation and recovering to a stable level when precipitation exceeded 500 mm. For mean temperature of the driest quarter (bio9), habitat suitability remained high between -40°C and 8°C, declining above 8°C. Habitat suitability also increased when precipitation seasonality (bio15) exceeded 20 (**Supplementary Figure 6**).

Overall, the four *Stipa* species exhibited distinct environmental response profiles, reflecting differentiated climatic dependencies across elevation, temperature regimes, and precipitation seasonality. These results highlight the species-specific ecological niches captured by the EM model and provide a robust foundation for subsequent analyses of distributional dynamics under future climate scenarios.

### 3.3 Current potential geographic distribution of four *Stipa* species

**Figure 2:**
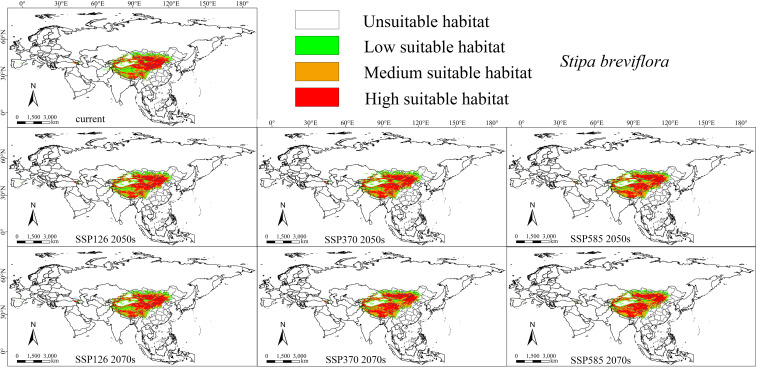
Potential distribution of *S. breviflora* under current and future different climate scenarios.

**Figure 3:**
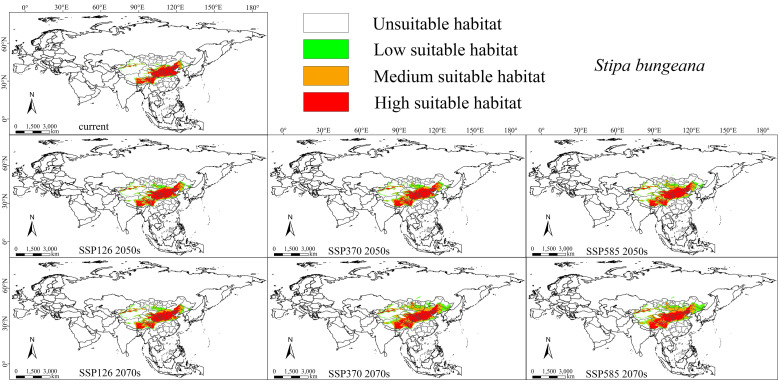
Potential distribution of *S. bungeana* under current and future different climate scenarios.

**Figure 4:**
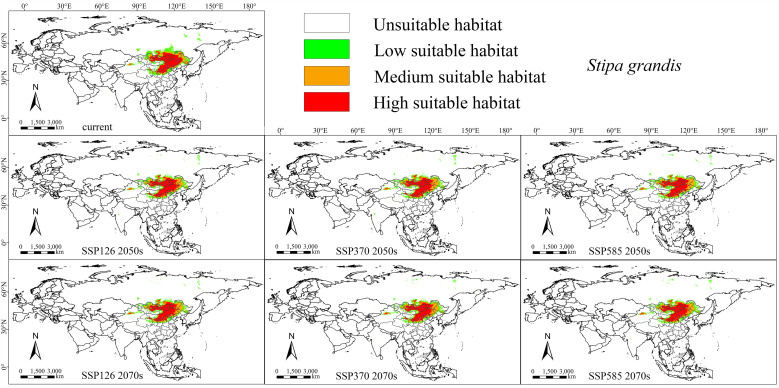
Potential distribution of *S. grandis* under current and future different climate scenarios.

**Figure 5:**
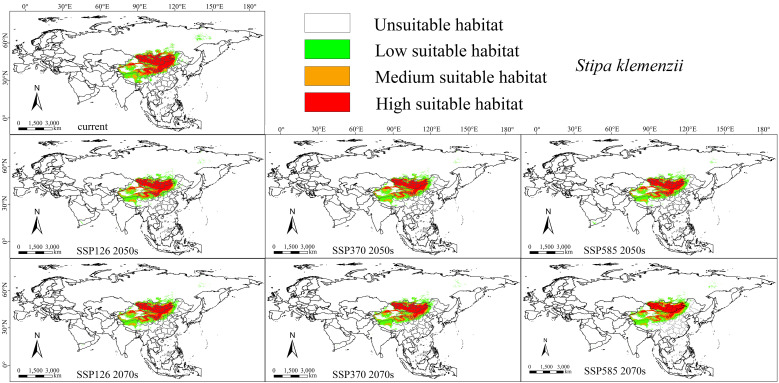
Potential distribution of *S. klemenzii* under current and future different climate scenarios.

Using the optimal ensemble model (EM), we estimated the current potential suitable habitats of *S. breviflora*, *S. bungeana*, *S. grandis*, and *S. klemenzii* (**Figures 2**–**5**; **Table 2**). Overall, the modelled spatial patterns correspond well with their known biogeographic ranges, supporting the reliability of the ensemble predictions. Although the four species showed broad spatial overlap across the (semi-) arid zones of Central Asia, substantial interspecies differences emerged in terms of total suitable area, suitability-level composition, and ecological niche preferences.

**Table 2:** Changes in suitable habitat areas of four *Stipa* species under different climate scenarios.

Species	Emission scenarios or periods	Suitable habitat (A/104 km2)			
		Low suitable habitat	Medium suitable habitat	High suitable habitat	Total suitable habitat
*S. breviflora*	current	219.98	154.08	280.12	654.18
	SSP126 (2050s)	195.99	174.82	292.67	663.48
	SSP126 (2070s)	196.81	176.82	292.52	666.15
	SSP370 (2050s)	185.02	191.12	294.93	671.07
	SSP370 (2070s)	159.38	206.17	294.96	660.51
	SSP585 (2050s)	171.96	195.46	294.93	662.35
	SSP585 (2070s)	160.68	210.37	293.36	664.41
*S. bungeana*	current	63.52	60.68	193.60	317.80
	SSP126 (2050s)	97.08	88.20	206.36	391.67
	SSP126 (2070s)	99.07	93.01	199.29	391.37
	SSP370 (2050s)	102.12	97.57	215.94	415.63
	SSP370 (2070s)	137.30	148.35	231.71	517.36
	SSP585 (2050s)	116.36	116.18	209.62	442.16
	SSP585 (2070s)	139.30	149.03	221.77	510.01
*S. grandis*	current	137.15	84.64	193.69	415.48
	SSP126 (2050s)	106.79	90.56	168.94	366.29
	SSP126 (2070s)	110.41	94.61	168.85	373.87
	SSP370 (2050s)	109.55	94.54	165.27	369.36
	SSP370 (2070s)	105.65	94.41	161.85	361.91
	SSP585 (2050s)	111.93	97.55	164.78	374.26
	SSP585 (2070s)	108.57	92.43	167.23	368.23
*S. klemenzii*	current	231.51	124.01	284.49	640.01
	SSP126 (2050s)	215.84	102.01	214.02	531.87
	SSP126 (2070s)	219.98	109.51	212.15	541.64
	SSP370 (2050s)	216.03	109.82	201.17	527.02
	SSP370 (2070s)	218.09	107.09	188.64	513.82
	SSP585 (2050s)	218.46	104.39	201.18	524.03
	SSP585 (2070s)	210.36	108.47	188.59	507.42

*Stipa breviflora*, a representative desert-steppe species, showed the widest potential distribution with a total suitable area of 654.18 × 10^4^ km^2^, characterized by a relatively balanced composition of high (280.12 × 10^4^ km^2^; 44.11%), medium (154.08 × 10^4^ km^2^; 26.35%), and low suitability habitat (219.98 × 10^4^ km^2^; 29.54%) (**Table 2**). High and medium suitability habitats are mainly concentrated in the central and southern parts of the Mongolian Plateau, the northwestern part of the Loess Plateau, the Tianshan Mountain, and the southeastern and northern outer edge mountains of the Tibetan Plateau such as the Kunlun Mountain, Altun Mountain, and Qilian Mountain in Central Asia, while low suitability habitats extended broadly into surrounding mountainous and semi-arid regions (**Figure 2**). Notably, the model predicted suitable areas in several regions lacking current natural distribution records (e.g., Armenia, Turkey, Spain, and central and northern Mongolia). This suggests that distributional limitations in this region may stem from ecological factors, such as niche vacancies resulting from mathematical extrapolation based on climatic similarity or limitations in species dispersal, as well as habitat fragmentation.

*Stipa bungeana* exhibited a high suitability in a concentrated region, with a total suitable area of 317.80 × 10^4^ km^2^. Among these, the high suitability area is dominant (193.60 × 10^4^ km²; accounting for 60.92% of the total area), and the medium suitability area is 60.68× 10^4^ km² (19.09%). The area of the low suitability habitat was 63.52× 10^4^ km² (19.99%) (**Table 2**). As a species adapted to warm and humid environments, the core areas of its high and medium suitability are mainly concentrated in southwestern Northeast China, parts of the North China Plain, the Loess Plateau, the southeastern Tibetan Plateau, and the Ili Valley. In contrast, low-suitability areas are distributed in the peripheral zones surrounding the medium- and high-suitability regions (**Figure 3**). Compared with the *S. breviflora*, this species prefers a relatively humid ecological environment.

*Stipa grandis*, a typical-steppe species, had a predicted suitable area of 415.48 × 10^4^ km², with high suitability habitat occupying 193.69 × 10^4^ km^2^ (46.62%), medium suitable habitat covering 84.64 × 10^4^ km^2^ (20.37%), and low suitable habitat accounting for roughly one-third (137.15 × 10^4^ km^2^; 33.01%) (**Table 2**). High and medium suitable habitats are primarily distributed across the eastern Mongolian Plateau, the northern part of the Loess Plateau, the northeastern part of the Tibetan Plateau, and the Tianshan Mountain, while low suitable habitat extended into southern and eastern Siberia and central and northeastern Mongolia (**Figure 4**). Although the model predicted suitable climatic conditions in the Russian Far East and Xinjiang, where natural populations have not been recorded, this discrepancy may reflect differences between the species’ environmental tolerance and its actual distribution range.

*Stipa klemenzii*, a desert-steppe species adapted to cold and arid conditions, had a total suitable area of 640.01 × 10^4^ km², with a suitability structure similar to that of *S. breviflora*. High suitable habitat accounted for 284.49 × 10^4^ km² (44.45%), medium suitable habitat for 124.01 × 10^4^ km² (19.38%), and low suitable habitat for 231.51 × 10^4^ km² (36.17%) (**Table 2**). Core habitats were concentrated across the Mongolian Plateau and northern Tibetan Plateau, while low suitable habitats extended into Buryatia, Zabaykalsky Krai, and the Tianshan-Kunlun region (**Figure 5**). This broad spatial extent reflects of *S. klemenzii* tolerance to arid environments and its capacity to survive under harsh climatic conditions.

Comparative analysis of the four *Stipa* species highlights both extensive overlap and clear ecological differentiation. *S. breviflora* and *S. klemenzii* shared the broadest potential ranges, reflecting their adaptation to relatively arid climates. Their distributions are shaped predominantly by low precipitation and strong temperature seasonality, allowing them to occupy large desert-steppe and plateau environments. By contrast, *S. bungeana* and *S. grandis* showed more constrained distributions and a higher dependence on mesic conditions. *S. bungeana* exhibited the strongest concentration of high suitable habitats, indicating a narrower ecological niche than the other species. *S. grandis* occupied an intermediate niche, overlapping substantially with *S. klemenzii* in the eastern Mongolian Plateau but showing a markedly greater affinity for relatively moist steppe environments.

### 3.4 Expansion and contraction under future climate scenarios

**Figure 6:**
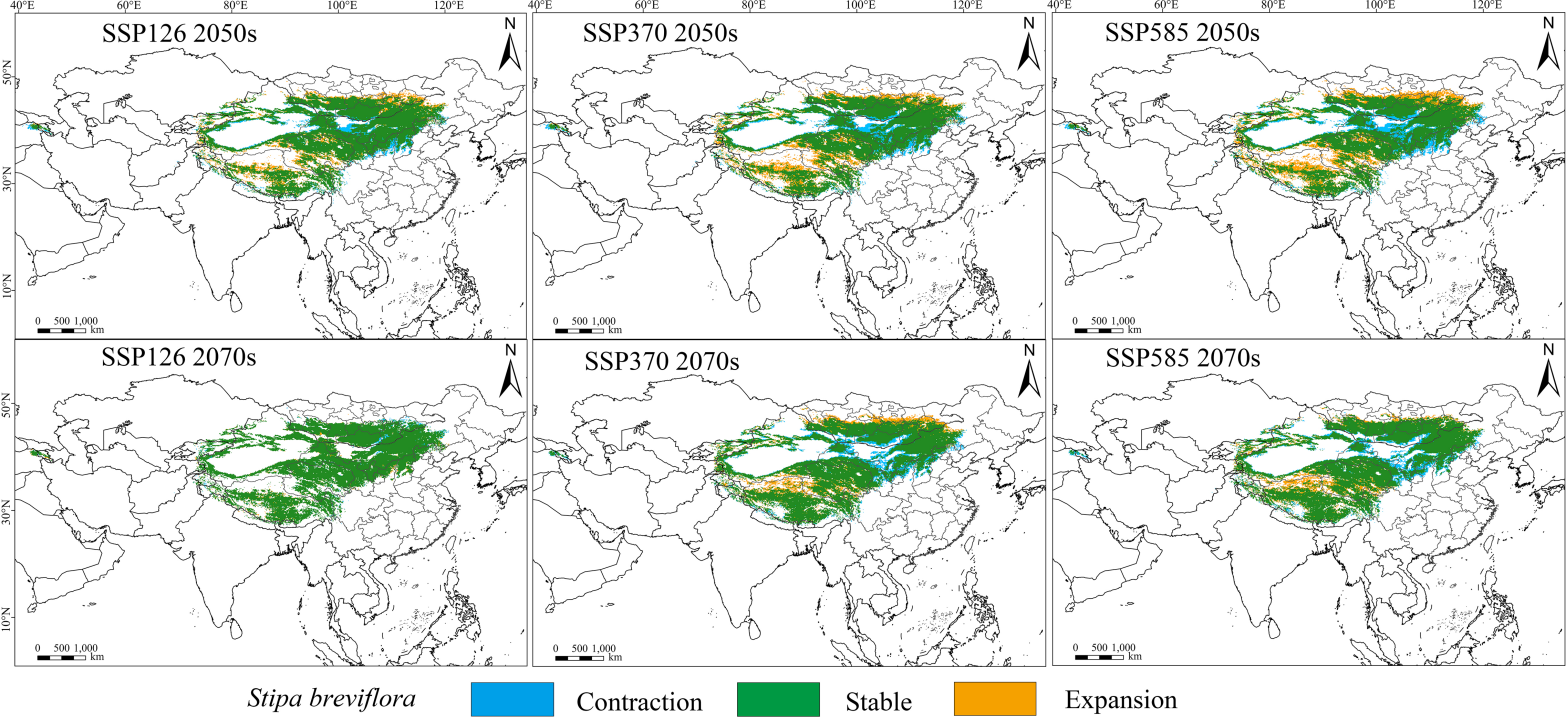
Changes in the suitable habitat distribution of *S. breviflora* under different climate scenarios.

**Figure 7:**
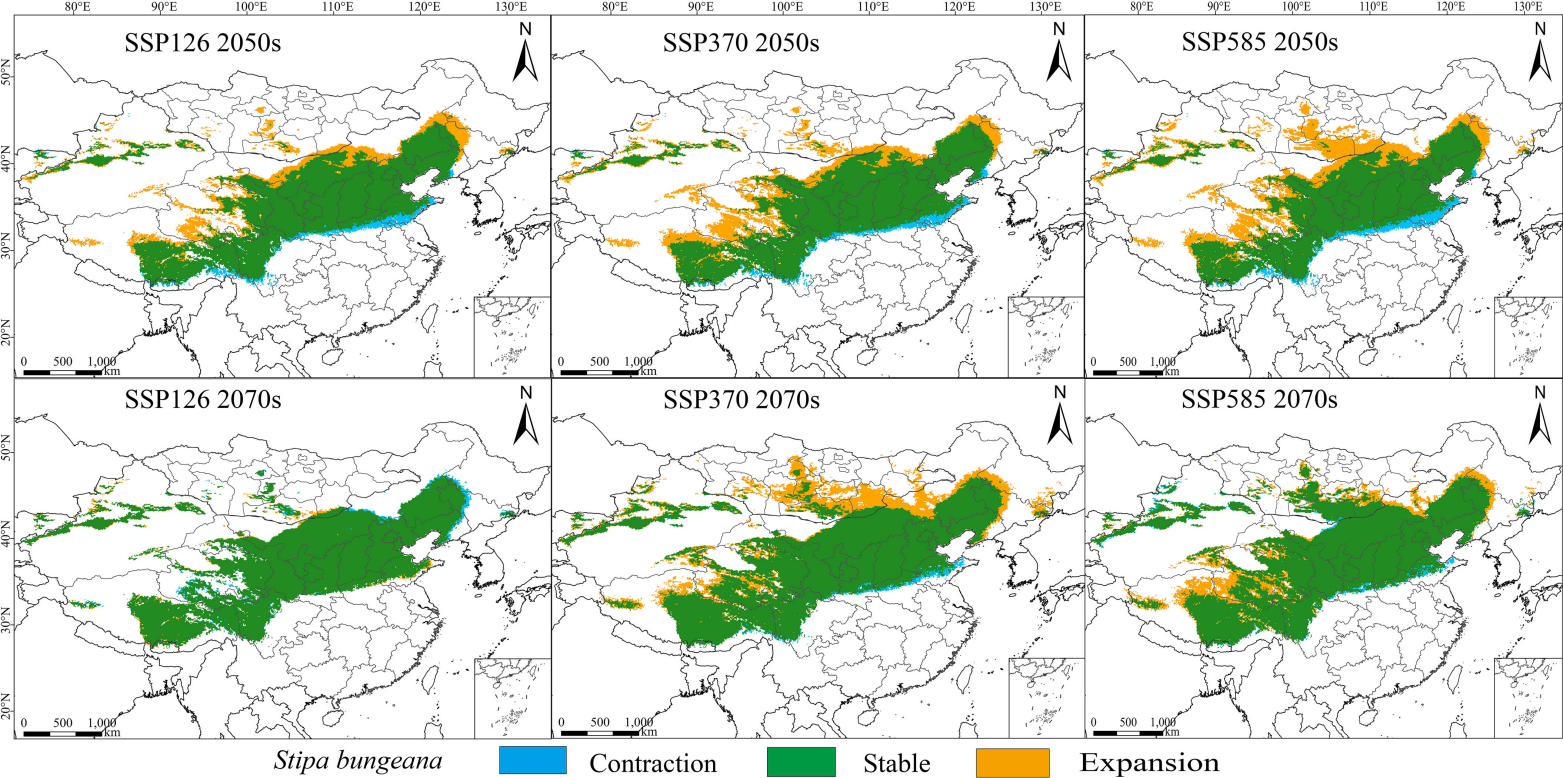
Changes in suitable habitat distribution of *S. bungeana* under different climate scenarios.

**Figure 8:**
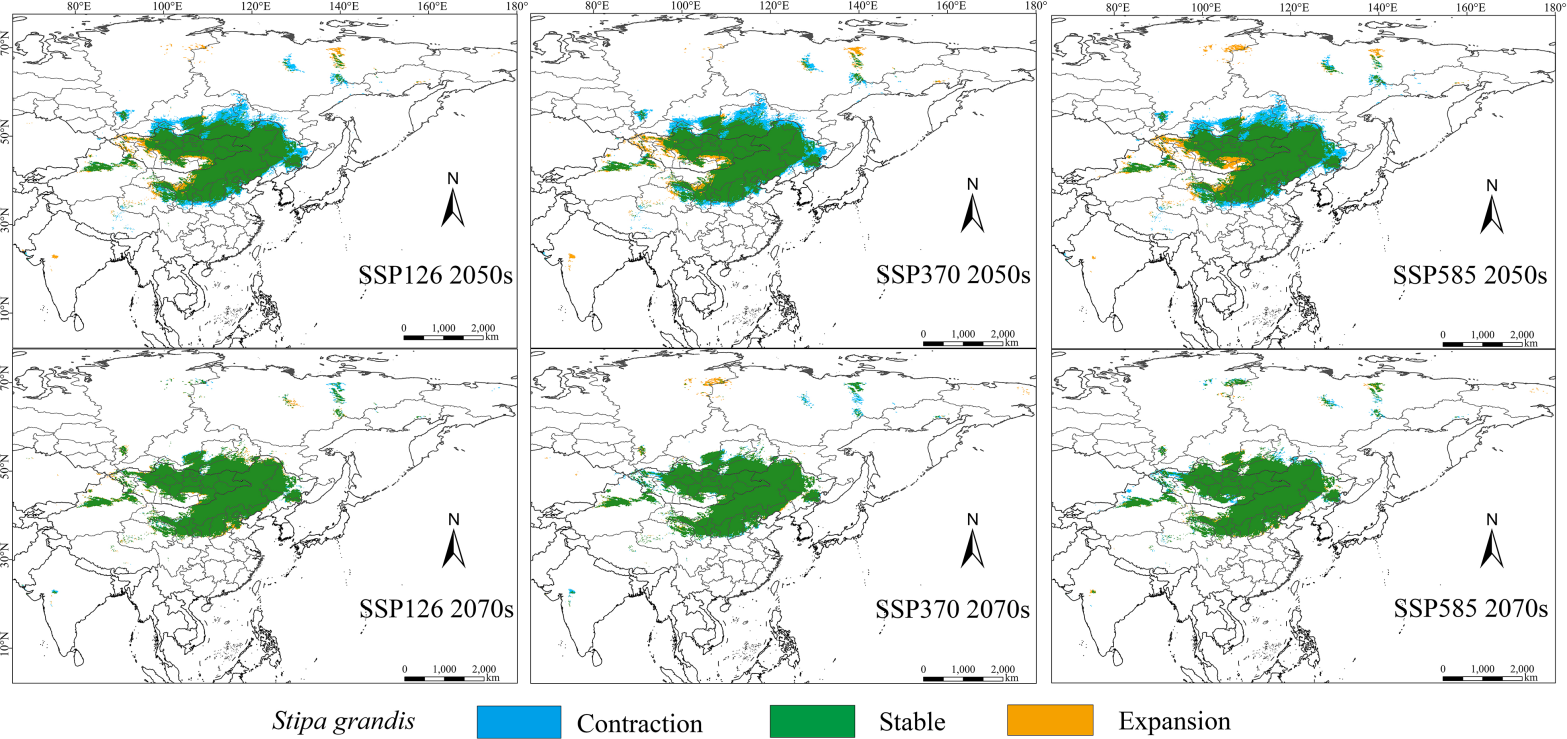
Changes in suitable habitat distribution of *S. grandis* under different climate scenarios.

**Figure 9:**
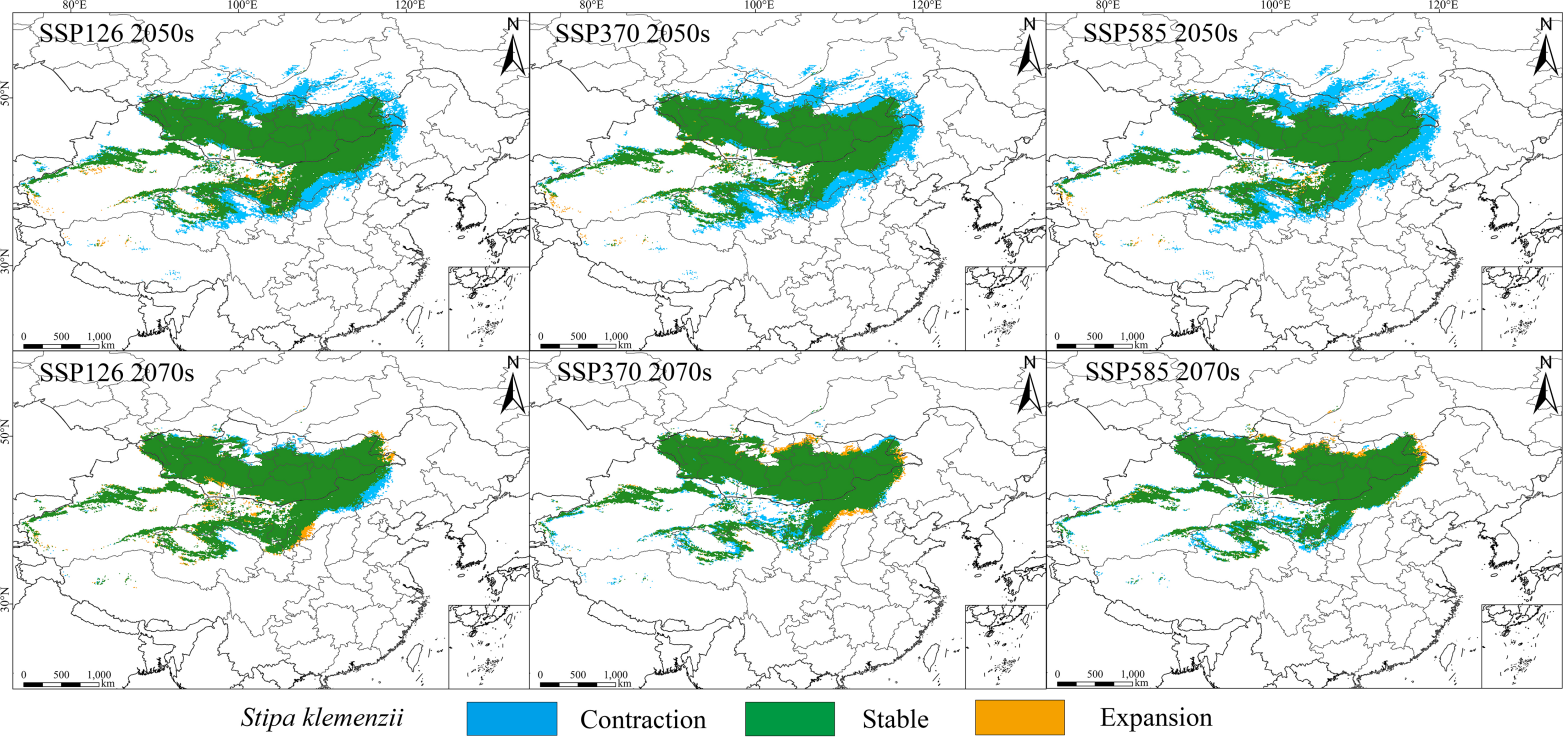
Changes in suitable habitat distribution of *S. klemenzii* under different climate scenarios.

Using the optimal ensemble model, we projected the potential future distributions of the four *Stipa* species for the 2050s and 2070s under three climate scenarios (SSP126, SSP370, and SSP585) (**Figures 2**–**9**; **Table 2**). Across all scenarios, the core suitable habitats of the species are expected to persist in Central Asia. However, the magnitude and spatial direction differ considerably among species.

**Figure 10:**
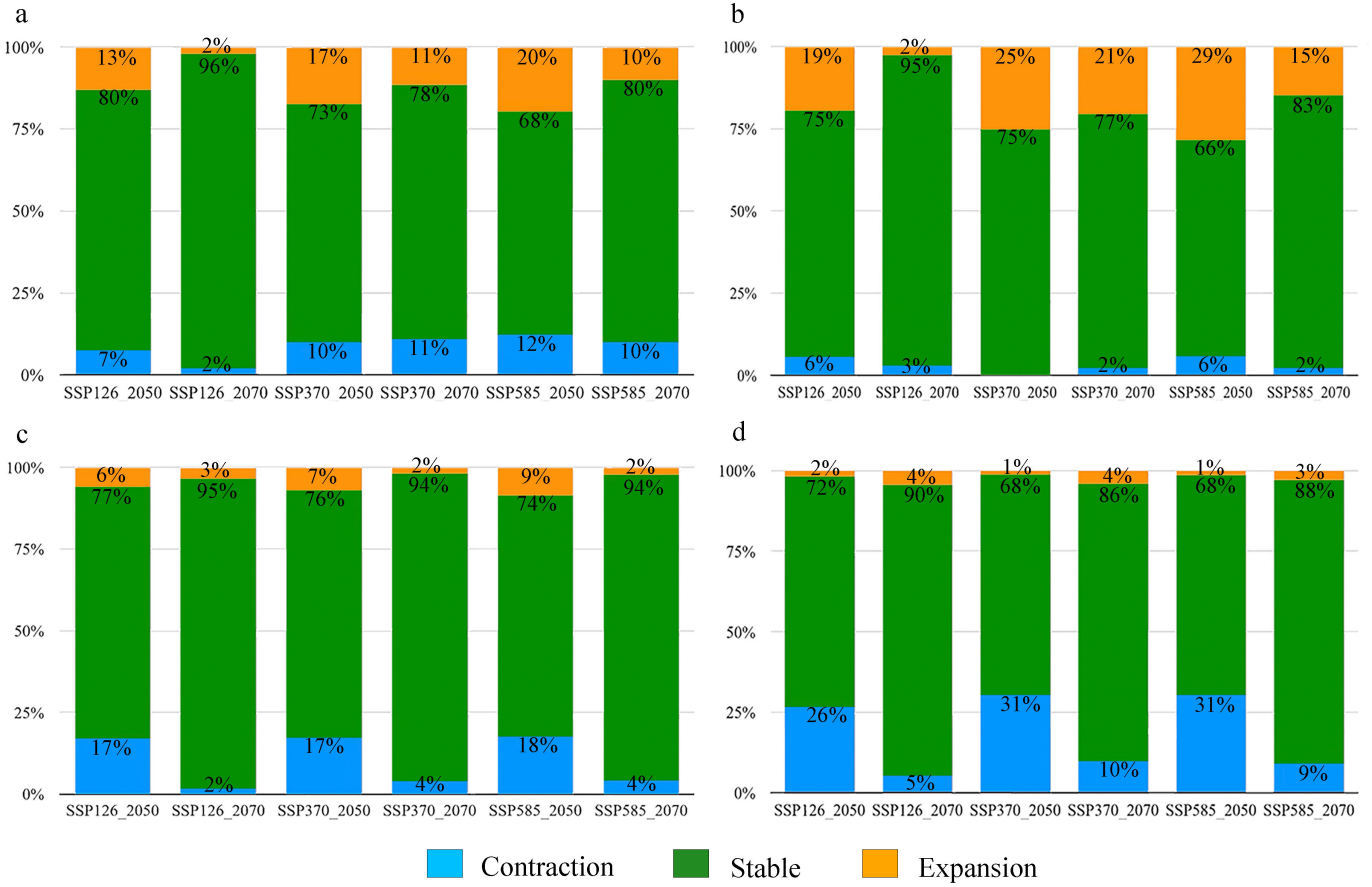
Proportions of contraction, expansion, and stable habitats of four *Stipa* species under future climate scenarios. **(a)** - *S. breviflora*, **(b)** - *S. bungeana*, **(c)** - *S. grandis*, **(d)** - *S. klemenzii*.

For *S. breviflora*, the future suitable habitat is projected to remain relatively stable across different climate scenarios (**Figure 2**), with fluctuation in its distribution during the 2050s. By the 2070s, the rates of habitat contraction and expansion are expected to slow down. Under the SSP126 scenario in the 2070s, the species’ habitat changes will be minimal. Overall, the species will likely maintain a relatively stable distribution pattern, showing low sensitivity to climate variables (**Figures 6**, **10a**).

For *S. bungeana*, suitable habitats are projected to expand significantly across future climate scenarios (**Figure 3**). As carbon emissions increase, its suitable habitat expands northwestward, reaching a total area of 391.37 × 10^4^ km^2^ (SSP126, 2070s), 517.36 × 10^4^ km^2^ (SSP370, 2070s), and 510.01 × 10^4^ km^2^ (SSP585, 2070s) (**Figure 3**; **Table 2**). Notably, under the SSP370 scenario, the total suitable area exceeds that of the SSP585 scenario, with a consistent northwestward expansion across all scenarios (**Figures 7**, **10b**).

In contrast, *S. grandis* is projected to experience an overall shrinking trend in its suitable habitat. Under all emission scenarios, its habitat is expected to decline to 373.87 × 10^4^ km^2^ under SSP126, 361.91 × 10^4^ km^2^ under SSP370, and 368.23 × 10^4^ km^2^ under SSP585 by the 2070s (**Figure 4**; **Table 2**). From the 2050s to the 2070s, the shrinkage rate of suitable habitats slowed down. (**Figures 8**, **10c**).

*Stipa klemenzii*, like *S. grandis*, is expected to show an overall decline in suitable habitats under future. By the 2070s, its habitat is projected to decrease to 541.64 × 10^4^ km^2^ under the SSP126 scenario, 513.82 × ^4^ km^2^ under the SSP370 scenario, and 507.42 × 10^4^ km^2^ under the SSP585 scenario (**Figure 5**; **Table 2**). From the 2050s to the 2070s, the shrinkage rate of suitable habitats slowed down (**Figures 9**, **10d**).

### 3.5 Centroid migration trajectories

**Figure 11:**
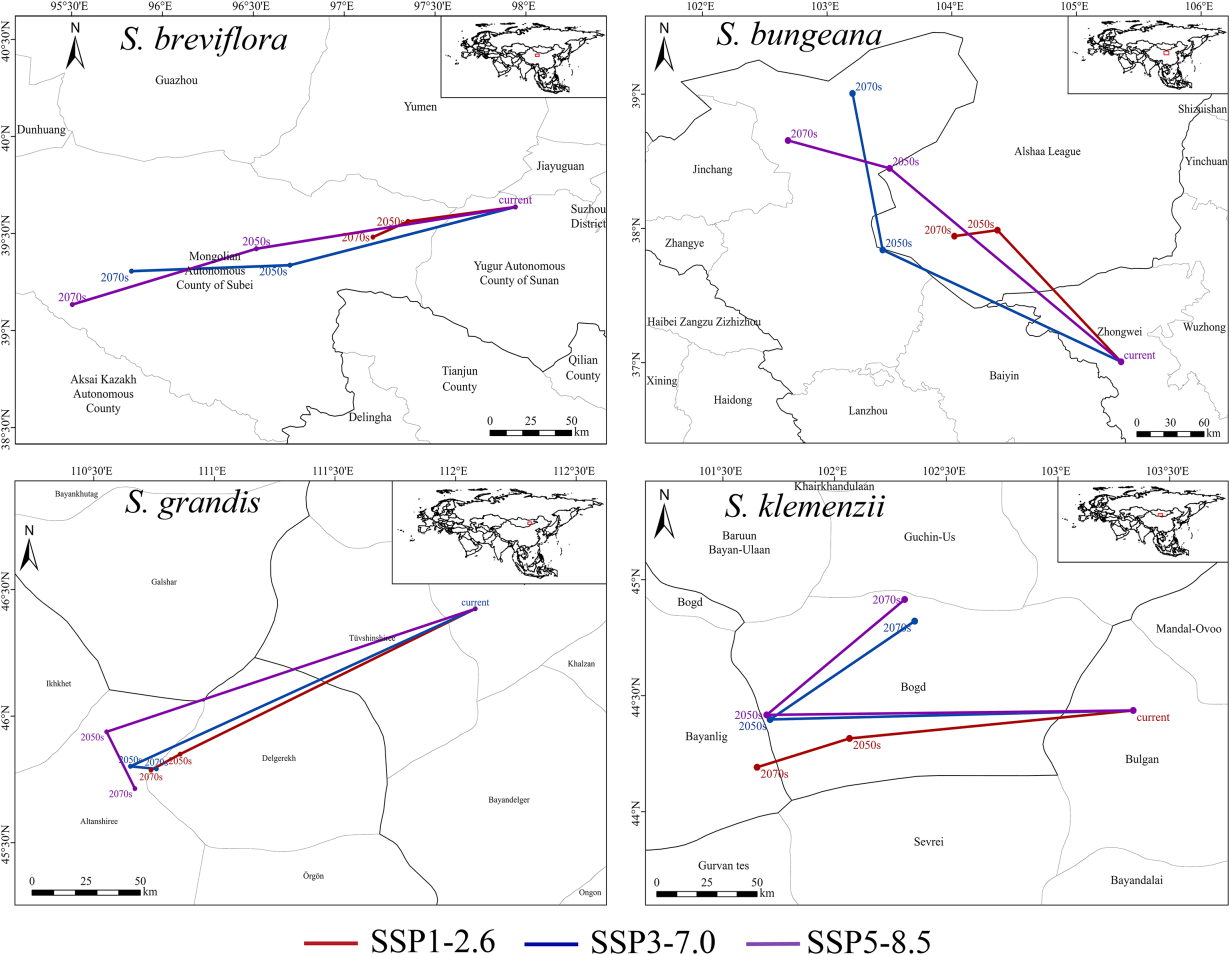
Centroid migration routes of suitable areas under different future climate scenarios.

Analyzing the centroid displacement reveals the migration trajectories of suitable habitats for the four *Stipa* species under future climate change (**Figure 11**). Currently, the potential distribution centroids of these four species are located in Central Asia (**Supplementary Table 6**). The centroids of the distribution centroids of all four *Stipa* species shifted westward. From the current to the 2050s, *S. breviflora* and *S. grandis* exhibit consistent southwestward migration trends across all three climate scenarios, whereas *S. bungeana* shows a northwestward migration over the same period. In contrast, *S. klemenzii* displays a scenario-dependent pattern, it migrates southwestward under the SSP126 scenario, but shifts westward under the SSP370 and SSP585 scenarios (**Figure 11**).

From the 2050s to the 2070s, the centroid trajectories vary markedly among species and across climate scenarios. Under the SSP126 scenario, *S. breviflora*, *S. grandis*, and *S. klemenzii* continue to migrate southwestward, whereas, *S. bungeana* shifts westward. Under the SSP370 scenario, migration directions diverge substantially, *S. breviflora* shifts westward, *S. grandis* migrates eastward, and *S. klemenzii* moves northeastward, and *S. bungeana* moves northwestward. Under the SSP585 scenario, *S. breviflora* migrates southwestward, *S. grandis* moves southeastward, and *S. klemenzii* continues to migrate northeastward, and *S. bungeana* moves northwestward (**Figure 11**).

The migration distance of the four species from the current to the 2050s, the migration distance is the shortest under the SSP126 scenario, and the migration distance increases with the increase of carbon emissions. In the SSP585 scenario, the longest migration distance was *S. bungeana*, 229.63 km, followed by *S. klemenzii*, 134.13 km, *S. grandis*, 131.63 km, and the shortest was *S. breviflora*, 129.90 km (**Supplementary Table 6**). From the 2050s to the 2070s, with the increase in carbon emissions, the migration distance of different species is also changing. Under the SSP585 scenario, the longest migration distance is *S. breviflora* 96.30 km, followed by *S. bungeana* 77.57 km, *S. klemenzii* 73.66 km, and *S. grandis* 26.11 km (**Supplementary Table 7**). Among the four species, *S. bungeana* exhibits the greatest overall centroid displacement, while *S. breviflora* exhibits the shortest (**Supplementary Table 7**), highlighting their relatively stronger spatial responses to future climate change.

## 4 Discussion

### 4.1 Climate factors determine the current distribution patterns

The geographical distribution pattern of *Stipa* species was driven by the matching of hydrothermal conditions and soil heterogeneity. Climatic factors played a leading role in species distribution and shaped the habitat preferences of different species. For temperature sensitive species, the distribution of *S. breviflora* was mainly affected by altitude, the mean temperature in the driest quarter (bio9), and solar radiation. This is different from the conclusion of Lv and Zhou (2018) that suggested that precipitation and temperature are the dominant factors. This difference may be related to variable selection and scale effects. *S. bungeana* was mainly affected by the mean temperature in the driest quarter (bio11) and precipitation of the coldest quarter (bio19), while Wang et al. (2019) suggested that it is mainly controlled by the temperature in the coldest quarter. This difference may be caused by differences in study areas, climate scenarios (such as RCP), and modeling methods (single model and integrated model), but both studies emphasize the important role of seasonal temperature.

For moisture-adapted species, the distribution of *S. grandis* was mainly influenced by precipitation of the coldest (bio19) and warmest quarter (bio18). This finding is consistent with the results of Zhang et al. (2025) on the *S. purpurea* community on the Tibetan Plateau, indicating that *S. purpurea*, like *S. grandis*, is highly sensitive to precipitation changes in both cold and warm seasons. This may be because the seasonal distribution of precipitation directly affects soil moisture, thereby limiting their germination, growth, and overwintering process. The results of this study differ from those of Tu (2020), who suggested that temperature seasonality (bio4) is the main factor affecting the distribution of *S. grandis*. This difference may be related to different settings of study areas and climate scenarios. Differences in hydrothermal conditions among regions can lead to changes in limiting factors. Meanwhile, the trends of climate variables also differ under different climate scenarios, such as RCP and SSP scenarios, which may affect the identification of dominant environmental factors by the model. *S. klemenzii* was mainly affected by precipitation of the coldest quarter (bio19) and mean temperature in the driest quarter (bio9). This result is highly consistent with the conclusion of Zhu et al. (2018) that emphasizes the importance of winter precipitation, such as precipitation in December (pre12), indicating that water supply during the non-growing season plays a key role in the distribution of *S. klemenzii*. This may be because winter precipitation indirectly regulates its growth process by affecting soil water storage and water availability on early spring.

Although *S. grandis* and *S. klemenzii* exhibited partial geographical overlap in the eastern Mongolian Plateau, their distribution differences may partly reflect ecological niche differentiation associated with heterogeneous habitat conditions. The occurrence of *S. klemenzii* in gravelly hills or saline-alkali environments could be associated with its relatively greater tolerance to harsh environmental conditions, which may contribute to its distribution stability.

### 4.2 Environmental drivers of future habitat expansion and contraction

Global climate change is reshaping the habitat distributions of *Stipa* species. The four *Stipa* species in Central Asia show divergent distributional responses under future climate scenarios. Under increasing carbon emissions, suitable habitats of the warm-adapted species *S. breviflora* and *S. bungeana* are projected to remain stable or expand. *S. breviflora*, owing to its strong physiological and ecological regulatory capacity, is expected to expand toward the Tibetan Plateau and central Mongolia, consistent with previously reported upward elevational shifts (Lv and Zhou, 2018). The distribution of *S. bungeana* was highly sensitive to climate warming and changes in precipitation patterns, with a clear expansion toward warmer zones. Warm-adapted species can adjust their physiological and ecological niches in response to environmental changes, enabling them to tolerate hydrothermal variability and enhance their adaptability (Lv et al., 2016; Lv and Zhou, 2018).

Different from the two warm-adapted species, the suitable habitats of *S. klemenzii* and *S. grandis*, which strongly depend on precipitation during the coldest season, are projected to decrease significantly under future climate scenarios. This pattern is consistent with the findings of Zhang et al. (2025), which showed that habitat suitability for these species declines markedly with increasing carbon emissions. The contraction of suitable habitats for these two species may be more closely associated with increasing temperature and altered moisture conditions under future climate change. In addition, rising temperatures and more frequent extreme weather events may shorten plant growth periods, thereby further reducing habitat suitability. Similar trends have also been reported in parts of Europe and Asia, where species sensitive to water and heat stress have adjusted their suitable habitat ranges in response to climate change (Duyar et al., 2025; Gan et al., 2025). The selective filtering effect driven by climate change may favor warm-adapted species while restricting cold-adapted species, thereby reshaping grassland community structure. Overall, species adapted to cold environments are expected to be more strongly affected by future climate change, although the rate of habitat contraction may gradually slow over time. Future changes in grassland types represented by *S. grandis* and *S. klemenzii* may profoundly influence grassland animal husbandry and the functioning of Eurasian grassland ecosystems in Central Asia. Therefore, grassland conservation and management strategies in Central Asia should be dynamically adjusted, with particular attention given to the contraction and migration trends of these species in order to enhance ecosystem stability and adaptive capacity.

### 4.3 Centroid migration patterns and their ecological implications

This study revealed strong scenario dependence and spatiotemporal heterogeneity in centroid migration among the four *Stipa* species. From the present to the 2050s, migration distances increase with stronger climate forcing from SSP126 to SSP585, confirming that species shift their distribution centroids under global warming to track stable climate niches (Parmesan and Yohe, 2003; Chen et al., 2011). In contrast, migration distances during the 2050s-2070s are lower than in the preceding period, suggesting potential constraints from physiological limits and dispersal barriers, and a transition toward restricted range dynamics under habitat fragmentation (Urban et al., 2016).

All four *Stipa* species exhibited a general westward shift in their distribution centroids under future climate scenarios. Temperature was one of the dominant factors shaping the predicted distribution boundaries, although additional ecological processes, such as interspecific competition and environmental filtering, may also influence future range dynamics (Tingley et al., 2009). The supplementary maps of the 2050s 30-year average annual temperature and precipitation under different SSP scenarios (**Supplementary Figures 7**, **8**) further support these westward shifts. Mean annual temperature in the core distribution regions increased consistently from SSP126 to SSP585, while precipitation patterns in Central Asia became increasingly unstable, potentially enhancing moisture limitation and reducing habitat suitability (Dai, 2013). These changes may be particularly important for relatively moisture-adapted species, whose distributions are strongly influenced by water availability. Under intensified warming and increasing aridity, eastern parts of the current range may become less suitable, whereas western regions may retain relatively favorable hydrothermal conditions, thereby promoting westward centroid shifts. This pattern highlights the ecological relevance of climate-driven redistribution in Central Asia grasslands (Pecl et al., 2017).

Under the SSP126 scenario, *S. breviflora*, *S. grandis*, and *S. klemenzii* exhibited southwestward shifts in their distribution centroids. This pattern may be associated with the persistence of relatively suitable hydrothermal conditions in southwestern regions under moderate climate warming. In arid and semi-arid ecosystems, species often track cooler and relatively moister habitats under future climate change, resulting in shifts toward environmentally favorable regions (Chen et al., 2011; Pecl et al., 2017). However, under the SSP370 and SSP585 scenarios, the migration directions of these species clearly diverged, suggesting species-specific differences in climate sensitivity and ecological adaptation under intensified warming (Chen et al., 2011). In contrast, the centroid of *S. bungeana* consistently shifted northwestward and toward higher latitudes under all three scenarios. This result is consistent with the view that species may respond differently to the same climate stress because of differences in physiological traits, dispersal capacity, and local environmental filtering (Lenoir and Svenning, 2015).

### 4.4 Conservation and management implications

Our findings provide important implications for grassland conservation and land management in Central Asia under future climate change. The projected shifts in suitable habitats and centroid migration of *Stipa* species suggest that climate change may substantially alter the structure and stability of semi-arid grassland ecosystems. Regions projected to remain suitable in the future may serve as potential climate refugia and should be prioritized for conservation and ecological restoration. From a management perspective, adaptive grazing management and long-term ecological monitoring should consider species-specific responses to future climate scenarios, particularly for climate-sensitive and cold-adapted species. In addition, the ensemble species distribution modeling framework applied in this study can be extended to other grassland plant species and ecologically vulnerable regions to support biodiversity conservation and sustainable ecosystem management under future climate change.

### 4.5 Model uncertainty, ensemble strengths, and limitations

Ensemble modeling effectively reduces prediction bias among individual algorithms through algorithmic complementarity, thereby improving robustness under complex climatic conditions (Wang et al., 2024; Yang et al., 2025; Pan et al., 2025). However, several sources of uncertainty remain in predicting the future distribution of *Stipa* species. In this study, future projections were primarily based on the BCC-CSM2-MR climate model, however, projections derived from a single GCM may still contain model-specific uncertainty. Differences in precipitation trends among climate models may particularly affect predictions for moisture-sensitive species such as *S. klemenzii* and *S. grandis.* To evaluate this uncertainty, we conducted a supplementary assessment using multiple CMIP6 GCMs, which showed high spatial consistency and low uncertainty across the projected suitable habitats (**Supplementary Figures 7–14**). The core suitable areas of the four *Stipa* species remained highly consistent among different climate trajectories, while uncertainty was mainly confined to marginal transition zones. These results suggest that the projected distribution shifts are primarily driven by consistent macroclimatic signals rather than variability among individual climate models. Nevertheless, incorporating additional multi-model ensemble projections could further improve prediction reliability.

Furthermore, due to the lack of reliable future soil datasets, this study assumed that current soil variables remain unchanged under future climate scenarios, which may introduce additional uncertainty into future habitat suitability projections. Another source of uncertainty arises from the use of default parameter settings across all modeling algorithms. Although this approach improved methodological consistency and comparability among species and algorithms, some machine-learning methods, such as RF, GBM, XGBOOST, and MAXENT, may achieve improved predictive performance under optimized parameter configurations. Future studies should therefore further evaluate the influence of parameter tuning on model accuracy and uncertainty under different climate scenarios.

In addition, the habitat suitability maps were categorized using an equal-interval classification scheme. Although this method facilitates comparison among species and future climate scenarios, it may not accurately represent species-specific ecological thresholds because suitability values are not necessarily distributed uniformly across taxa and models. Consequently, some marginal habitats may be overestimated or underestimated within particular suitability classes. Future studies could further evaluate threshold-based approaches, such as maximum TSS, maximum sensitivity plus specificity, or training-presence thresholds, to improve the ecological interpretability of habitat suitability classification.

Beyond classification uncertainty, existing environmental screening frameworks often neglect important biological and anthropogenic factors, including interspecific competition, land-use change, and grazing disturbance. In particular, the simplification of dispersal constraints may lead to an overestimation of species colonization capacity in fragmented landscapes. Therefore, although ensemble models have become important tools for assessing species responses to climate change, their ecological inference capacity remains constrained by the simplification of complex ecological processes. Future studies should further integrate biological interactions, dispersal mechanisms, and dynamic environmental processes into modeling frameworks to improve prediction accuracy and provide more practical scientific support for the precise conservation and sustainable management of grassland ecosystems.

## 5 Conclusions

Based on the Biomod2 model, this study indicates that the four constructive species of the genus *Stipa* in Central Asia exhibited significantly differentiated distribution responses to future climate change. The roles of different environmental factors in driving the distribution changes of different species varied significantly. *S. breviflora* was mainly affected by elevation, mean temperature during the driest quarter and solar radiation. *S. bungeana* was jointly driven by mean temperature and precipitation during the coldest quarter and precipitation in the warmest quarter. *S. grandis* was affected by precipitation during the coldest and warmest quarter and the temperature seasonality. *S. klemenzii* was more sensitive to seasonal changes in precipitation in the coldest quarter, mean temperature of the driest quarter, and precipitation seasonality. Overall, the centroids of the four *Stipa* species all shifted westward. The suitable habitats of *S. breviflora* and *S. bungeana*, which are adapted to warm environments, remained stable or expand. Among them, *S. breviflora* migrated southwestward and showed strong adaptability, while *S. bungeana* significantly expanded northwestward and was highly sensitive to changes in temperature and precipitation. However, the suitable habitats of *S. grandis* and *S. klemenzii*, which are adapted to cold environments, shrunk significantly. *S. grandis* migrated southwestward, while the migration direction of *S. klemenzii* changed from westward to northeastward with the increase of carbon emissions and time periods, indicating a more complex response. Overall, species adapted to cold environments will be more strongly affected by future climate change. Therefore, in the management and ecological restoration of grasslands in Central Asia, priority should be given to their distribution contraction and migration trends in order to maintain ecosystem stability.

## Data Availability

The datasets presented in this study can be found in online repositories. The names of the repository/repositories and accession number(s) can be found in the article/**Supplementary Material**.
