# Supplementary material for: Climate-driven habitat shifts reveal contrasting climate association of four Stipa species in Central Asia
Source: Front Plant Sci. 2026 Jun 24;17:1815614. doi: 10.3389/fpls.2026.1815614 (PMC13347222; doi:10.3389/fpls.2026.1815614)
Supplement: Supplementary file 1 [file DataSheet1.docx]

Supplementary Material

# Supplementary Figures and Tables

## Supplementary Figures


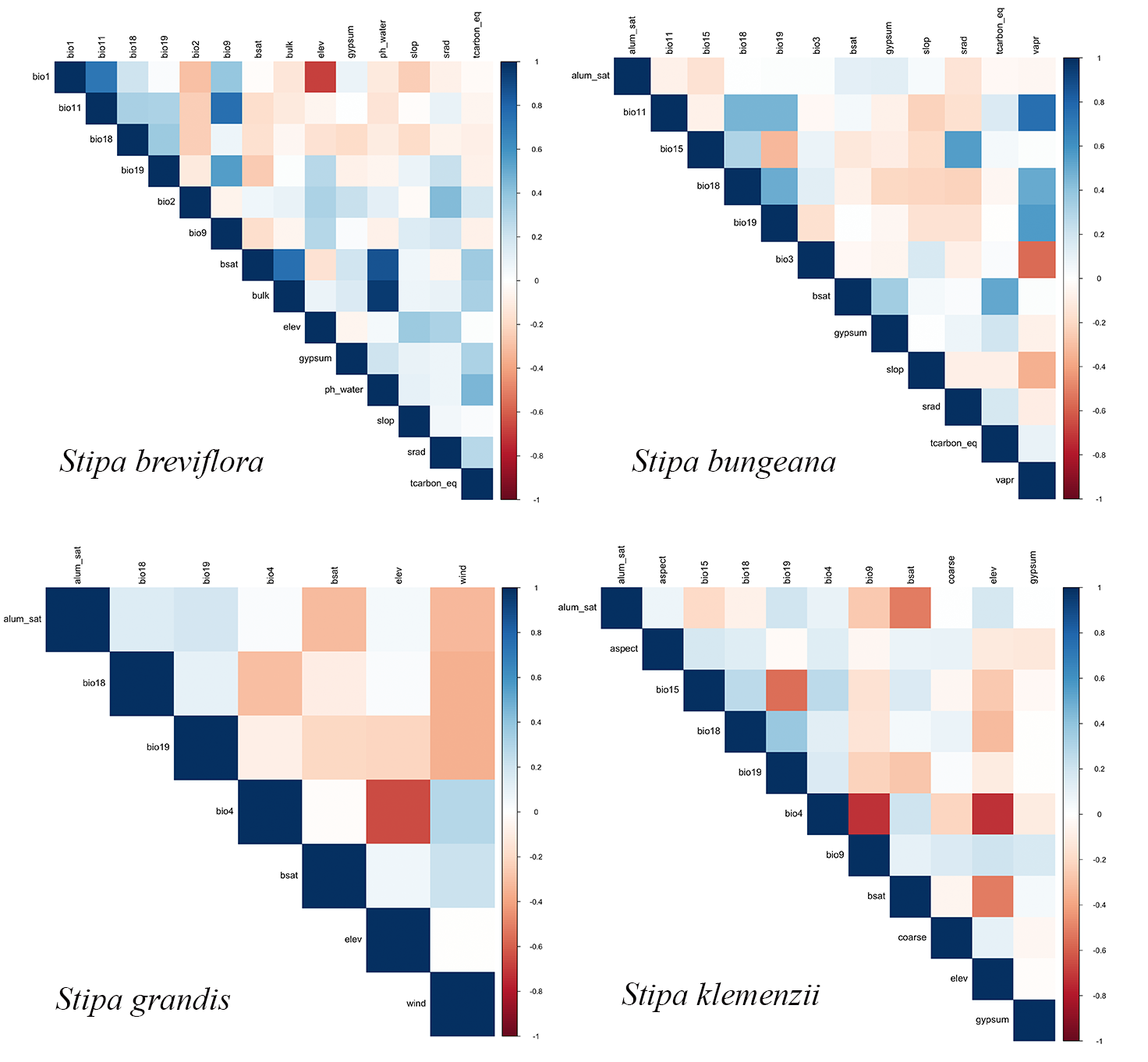


**Figure S1.** Correlation heat maps of 4 species of *Stipa.* (blue indicates a positive correlation, red indicates a negative correlation).


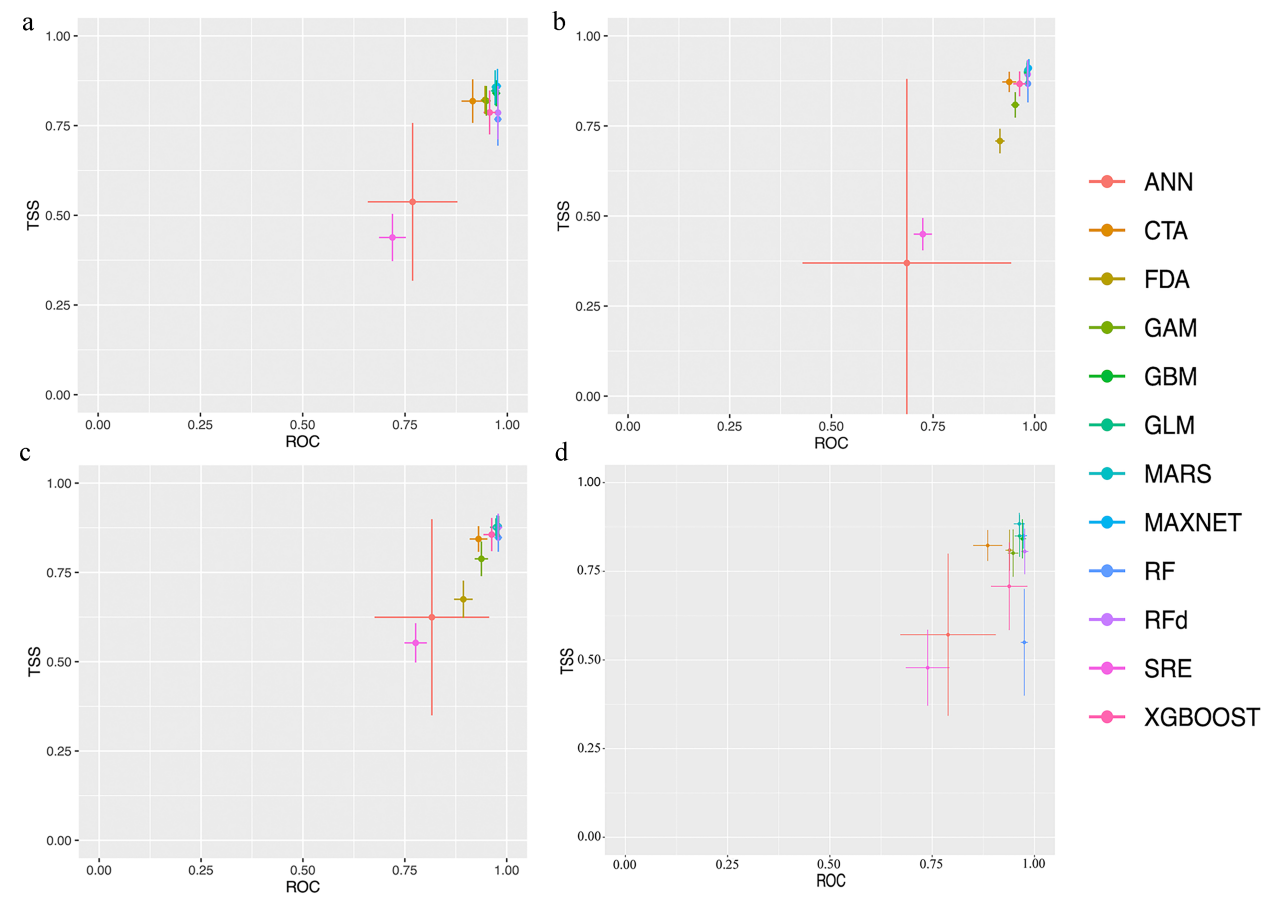


**Figure S2**. Average ROC and TSS Values for four *Stipa* Species. a. - *S. breviflora*, b. - *S. bungeana*, c. - *S. grandis*, d. - *S. klemenzii*.

**Note**: ANN, Artificial Neural Network; CTA, Classification Tree Analysis; FDA, Flexible Discriminant Analysis; GAM, Generalized Additive Model; GBM, Generalized Boosting Model; GLM, Generalized Linear Model; MARS, Multivariate Adaptive Regression Splines; MAXENT, Maximum Entropy; RF, Random Forests; RFd, Random Forest downsampled; SRE, Surface Range Envelope; XGBOOST, eXtreme Gradient Boosting Training. Model evaluation metrics include AUC, Area Under the Receiver Operating Characteristic Curve; and TSS, True Skill Statistic.


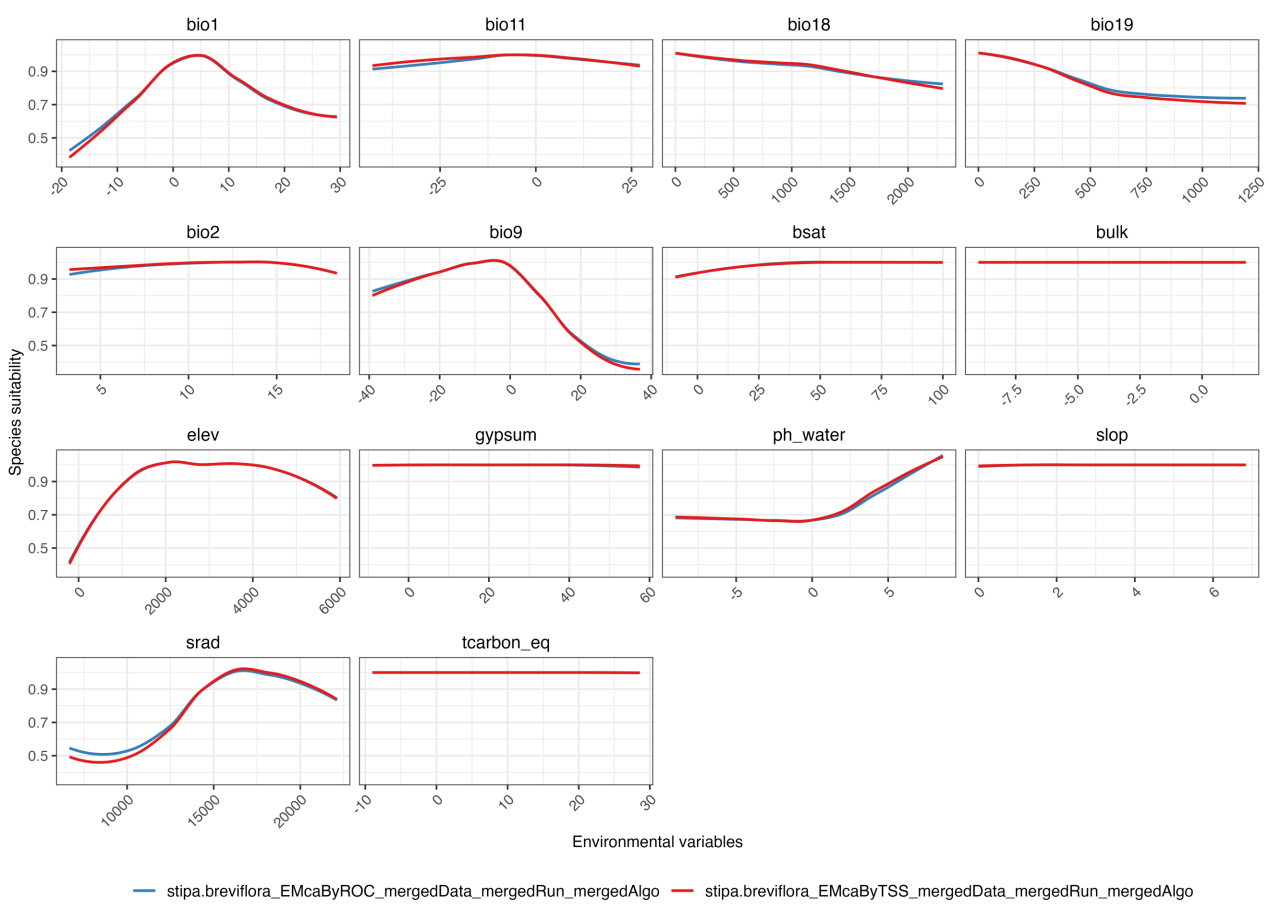


**Figure S3**. Response curve of *Stipa breviflora.*

*
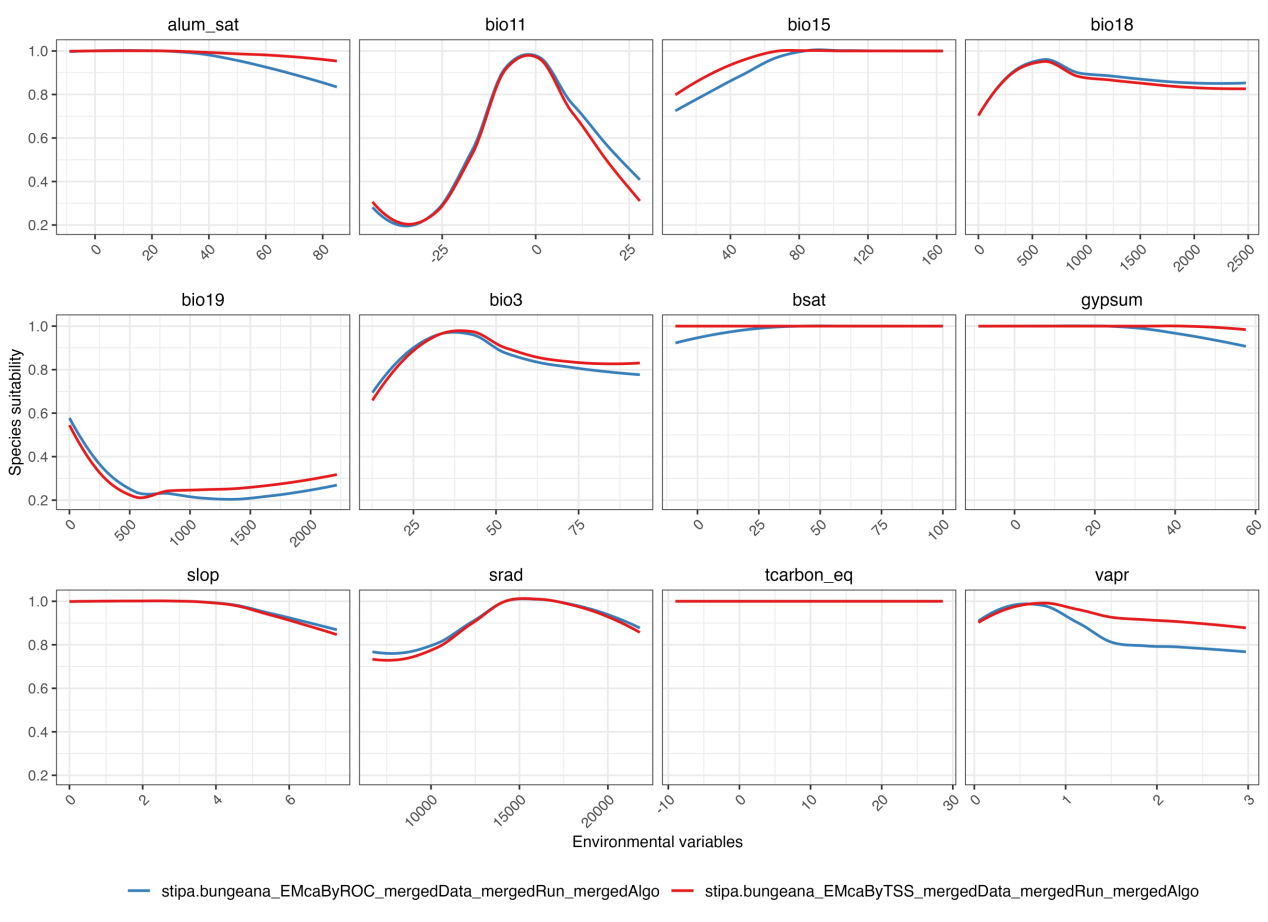
*

**Figure S4**. Response curve of *Stipa bungeana.*

*
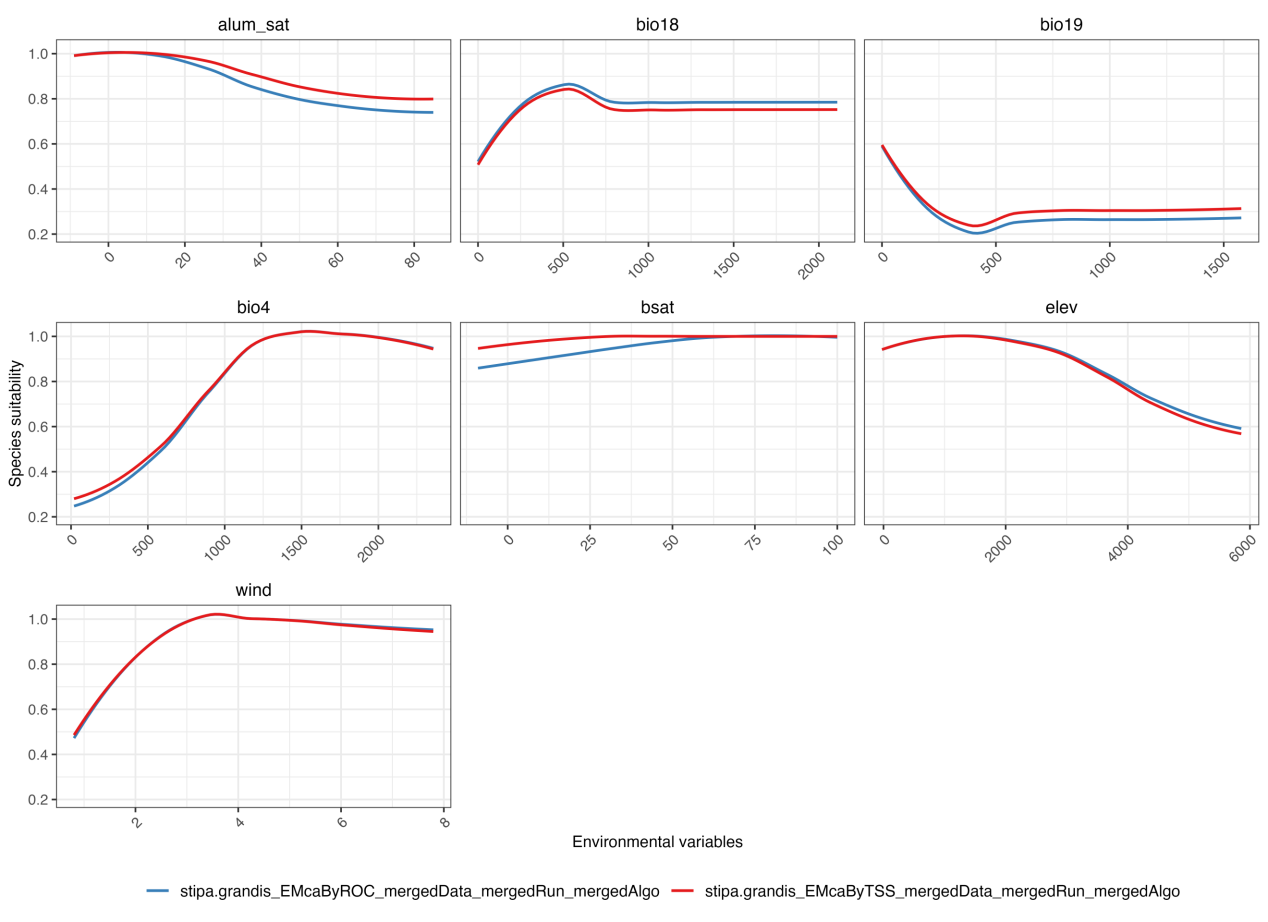
*

**Figure S5**. Response curve of *Stipa grandis*.

*
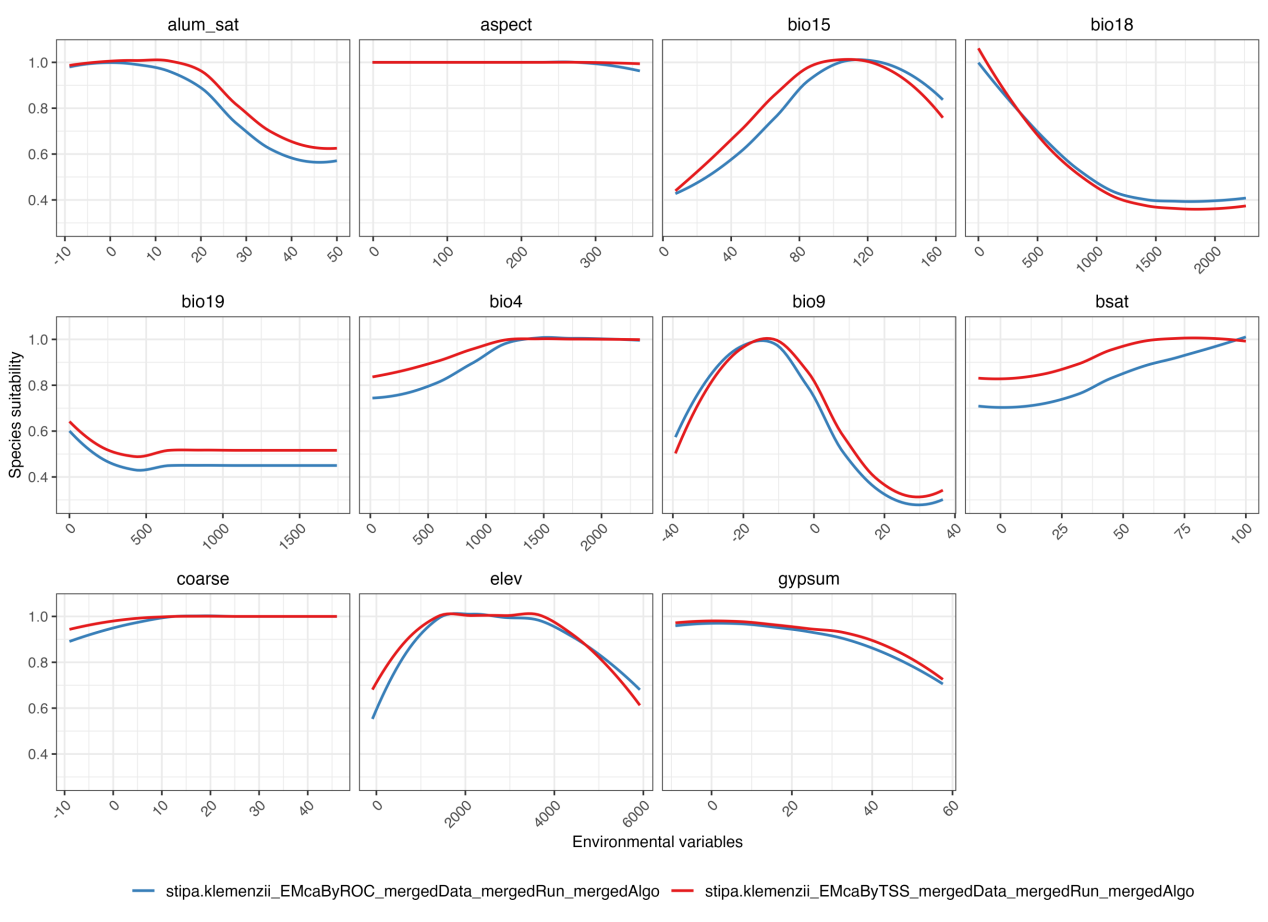
*

**Figure S6**. Response curve of *Stipa klemenzii.*


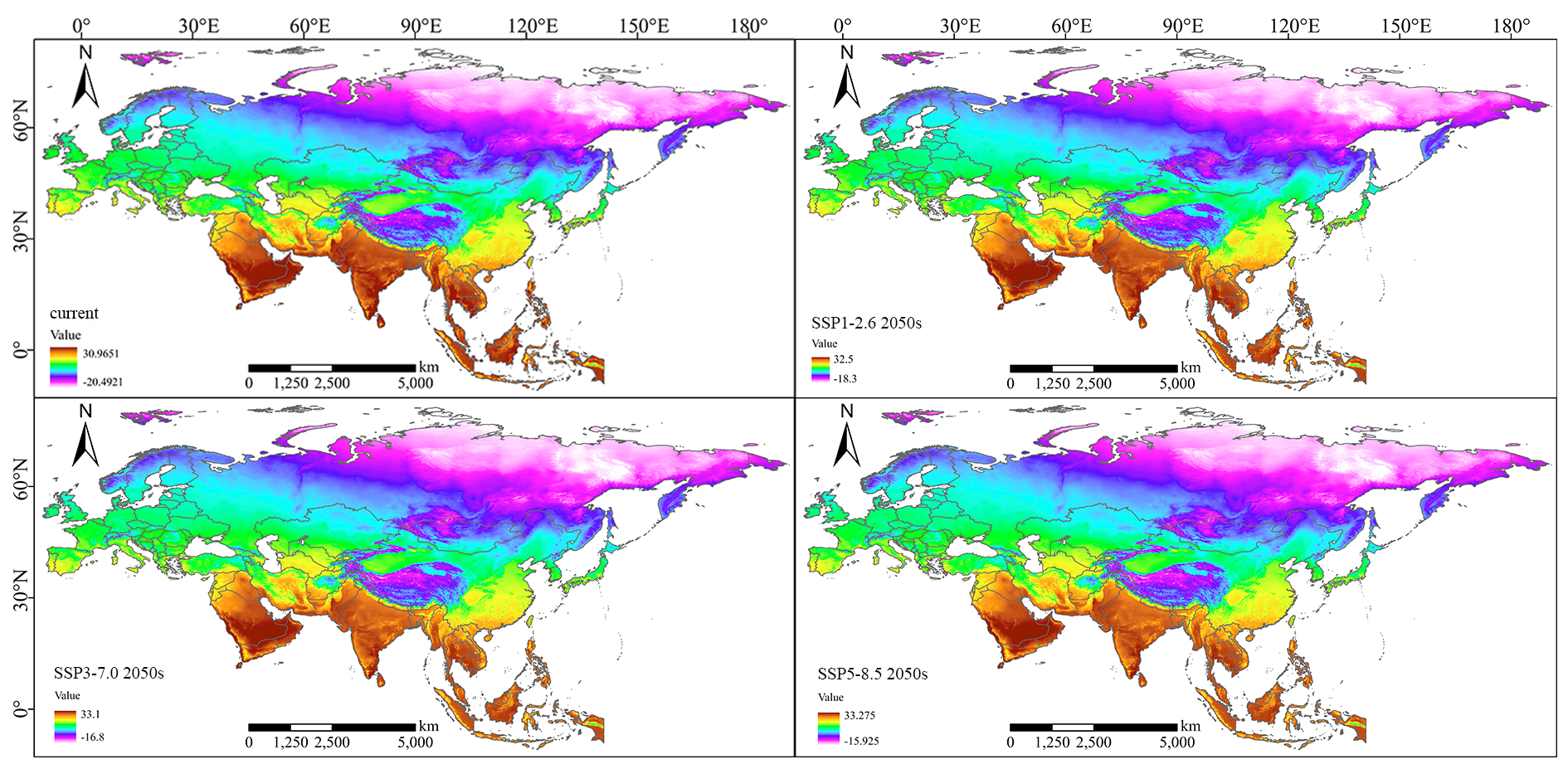


**Figure S7**. Average annual temperature under different scenarios in the 2050s.

**
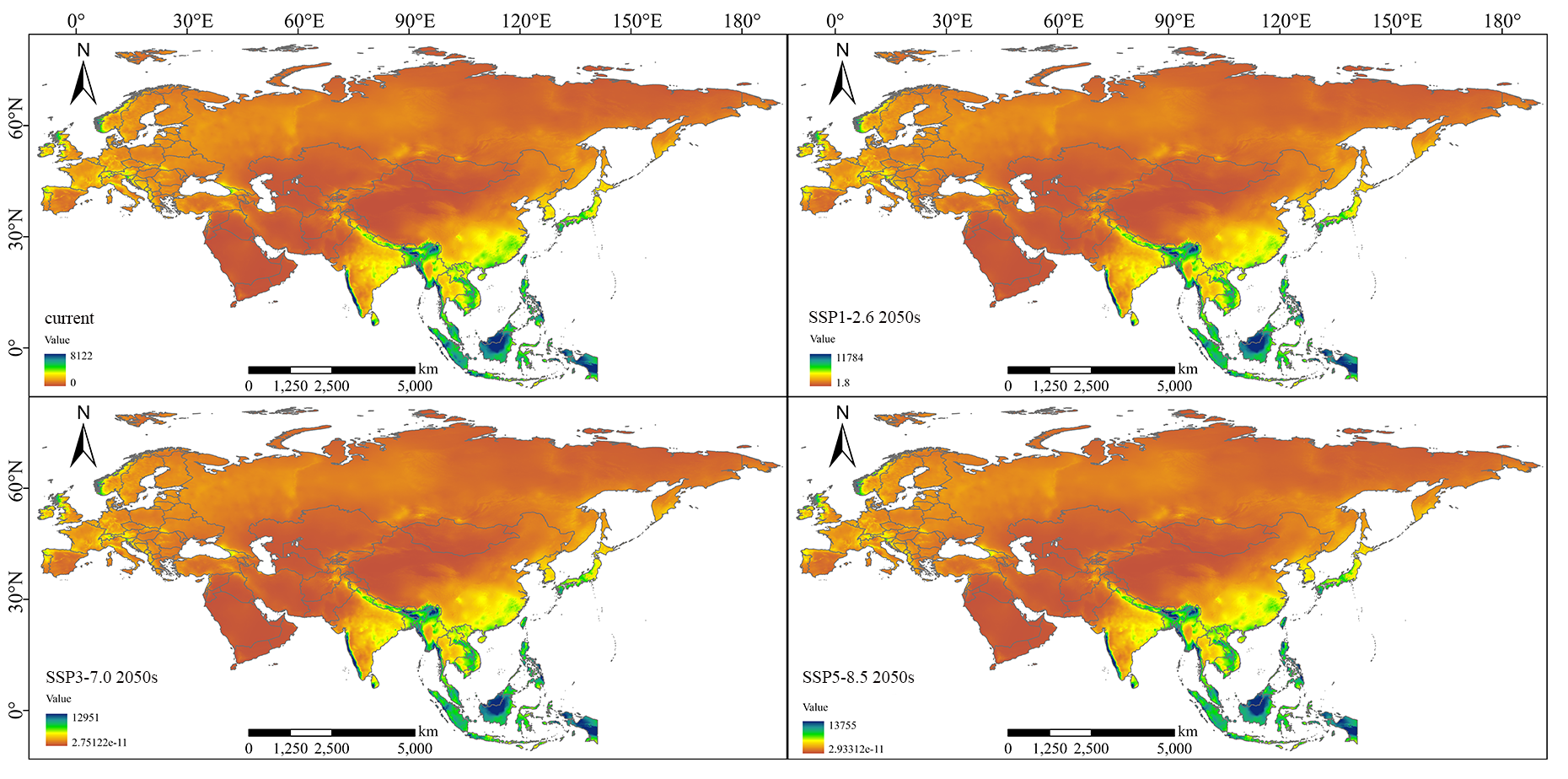
**

**Figure S8**. Average annual precipitation under different scenarios in the 2050s.


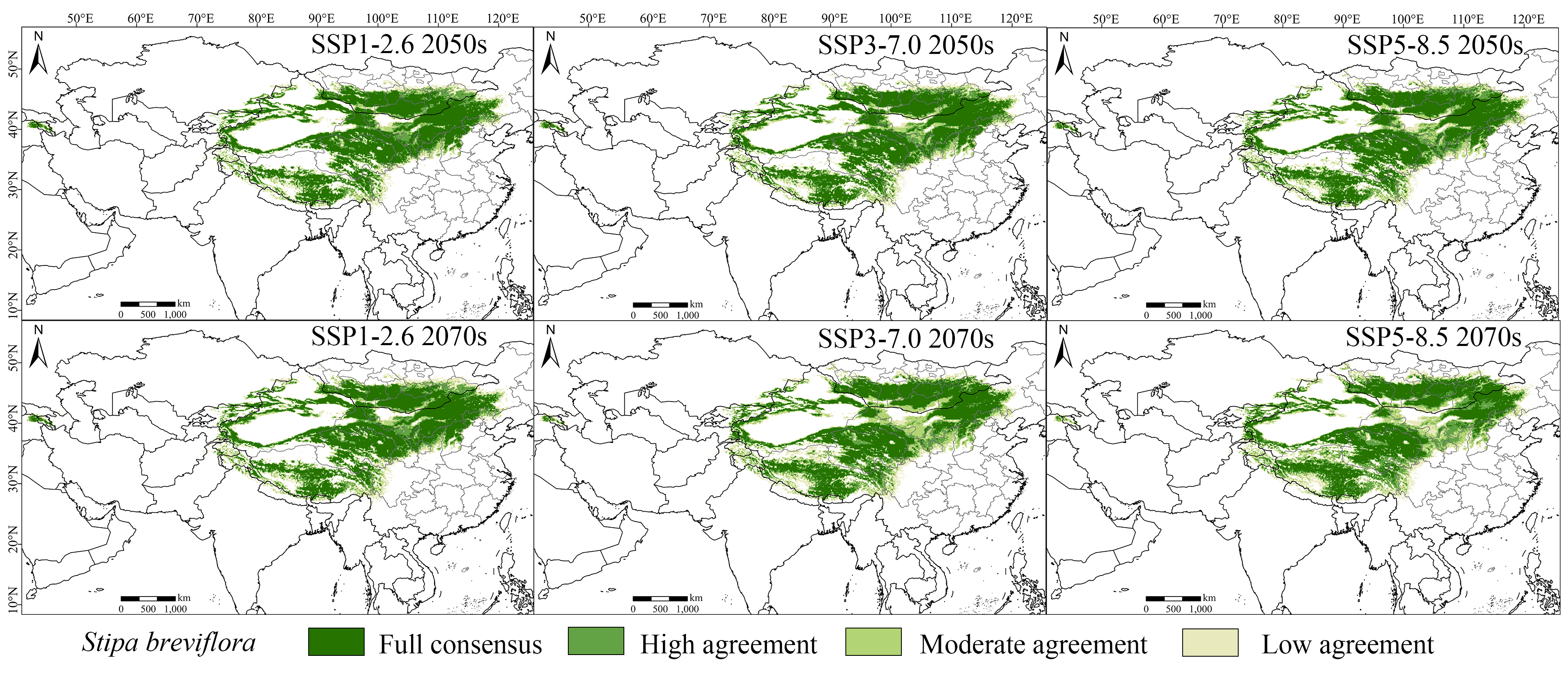


**Figure S9**. Consensus map of *Stipa breviflora* in different time periods and different scenarios.


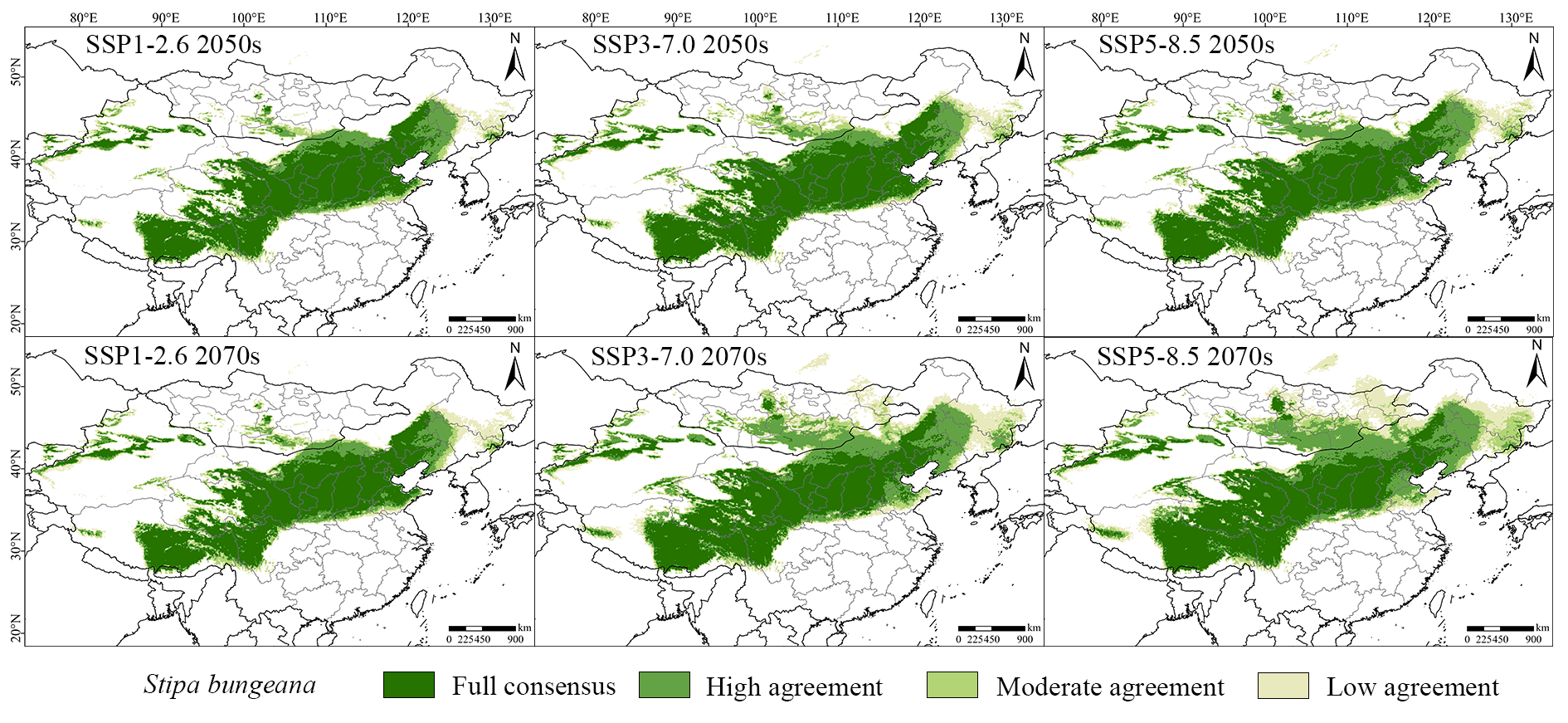


**Figure S10**. Consensus map of *Stipa bungeana* in different time periods and different scenarios.


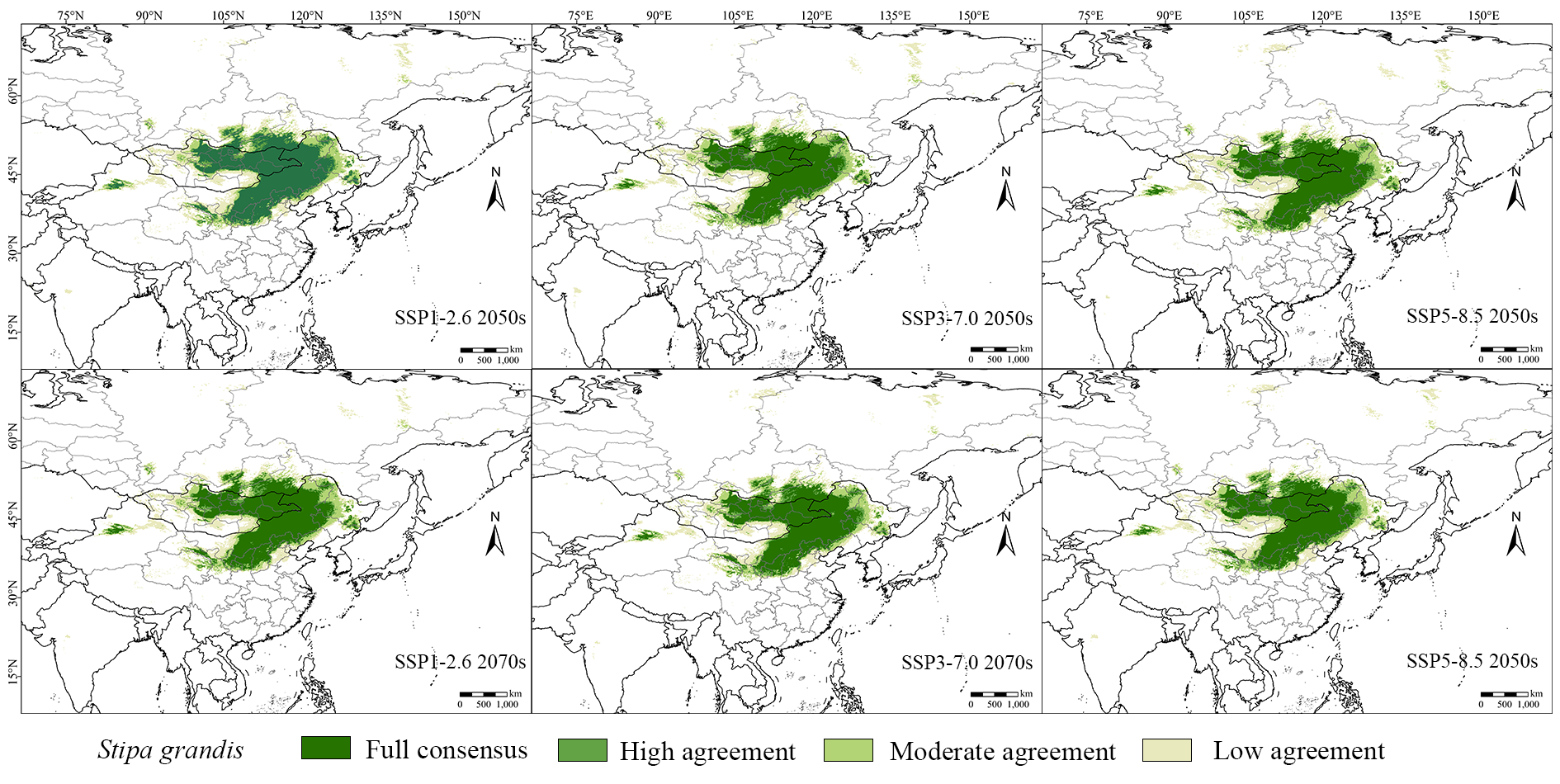


**Figure S11.** Consensus map of *Stipa grandis* in different time periods and different scenarios.


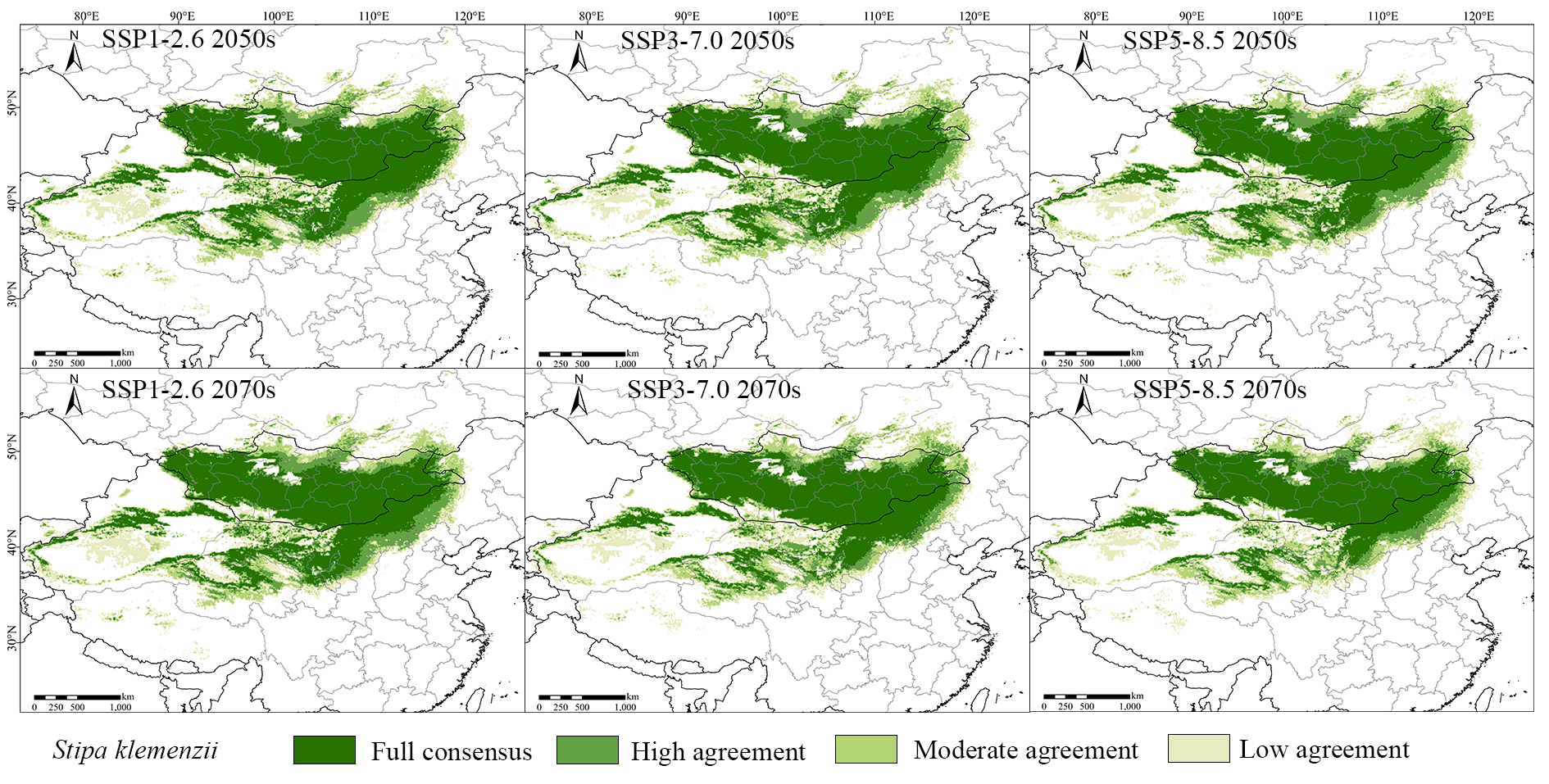


**Figure S12.** Consensus map of *Stipa klemenzii* in different time periods and different scenarios.


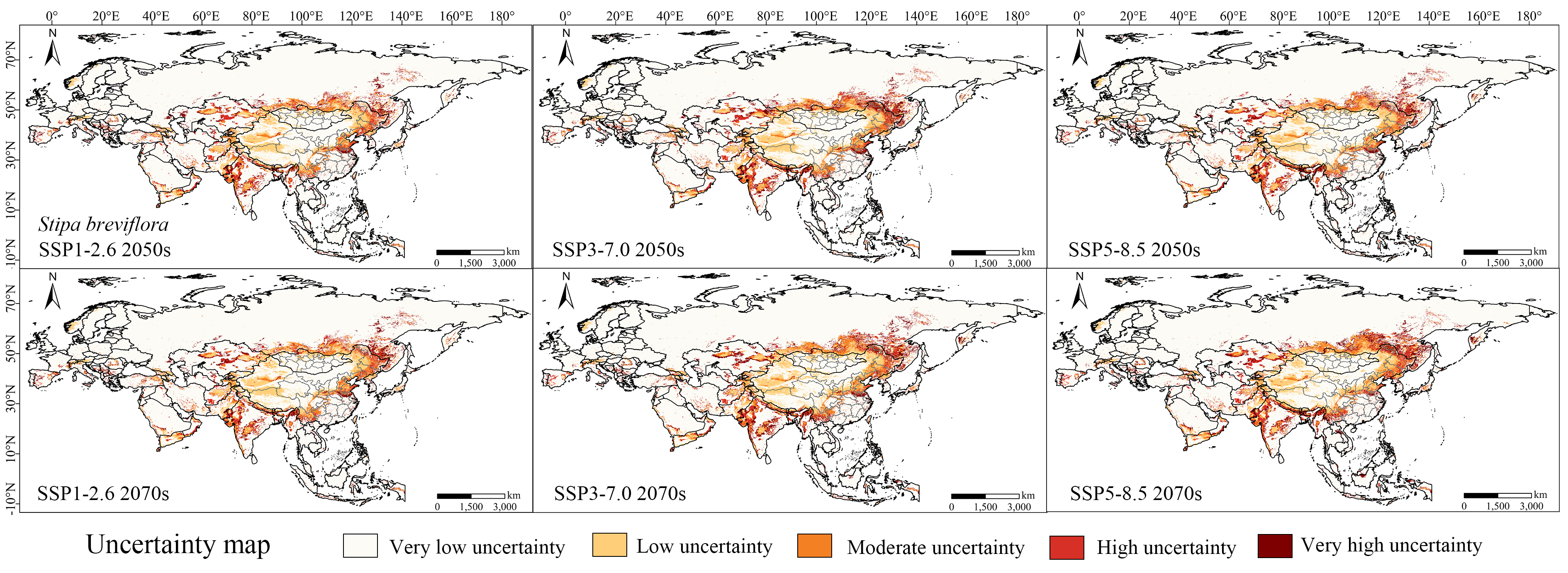


**Figure S13**. The uncertainty map of *Stipa breviflora* in different time periods and different scenarios.


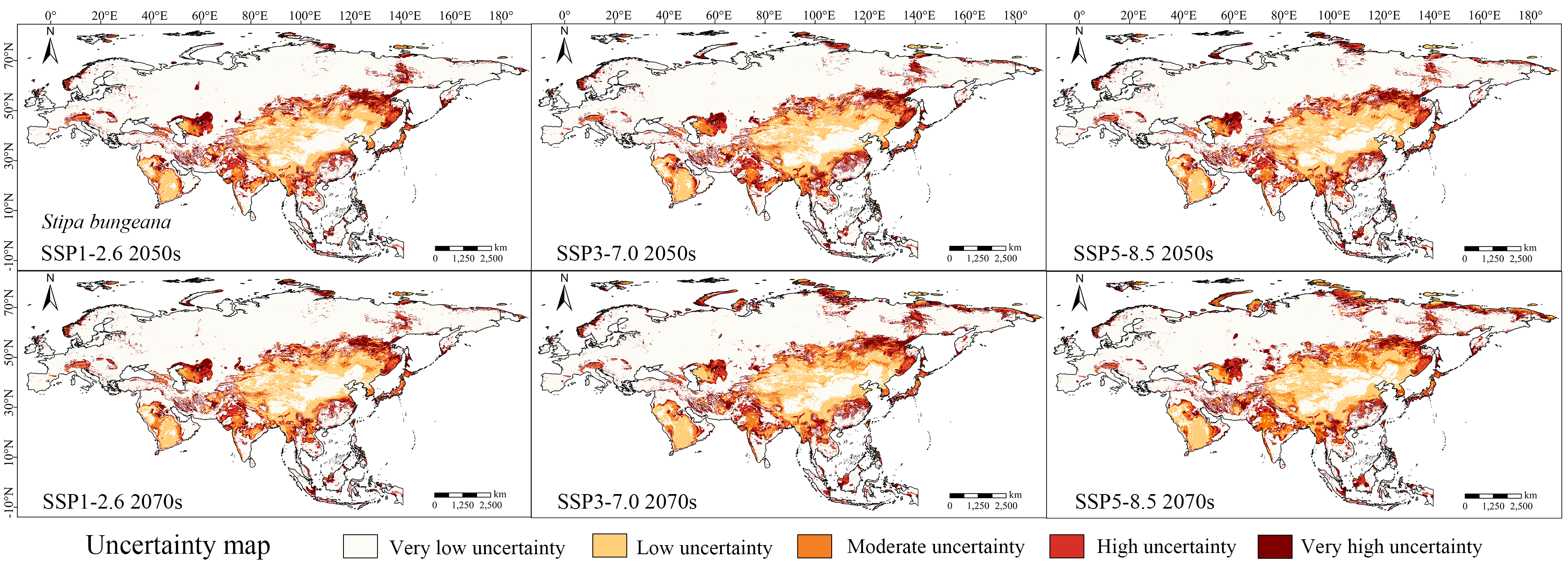


**Figure S14**. The uncertainty map of *Stipa bungeana* in different time periods and different scenarios.


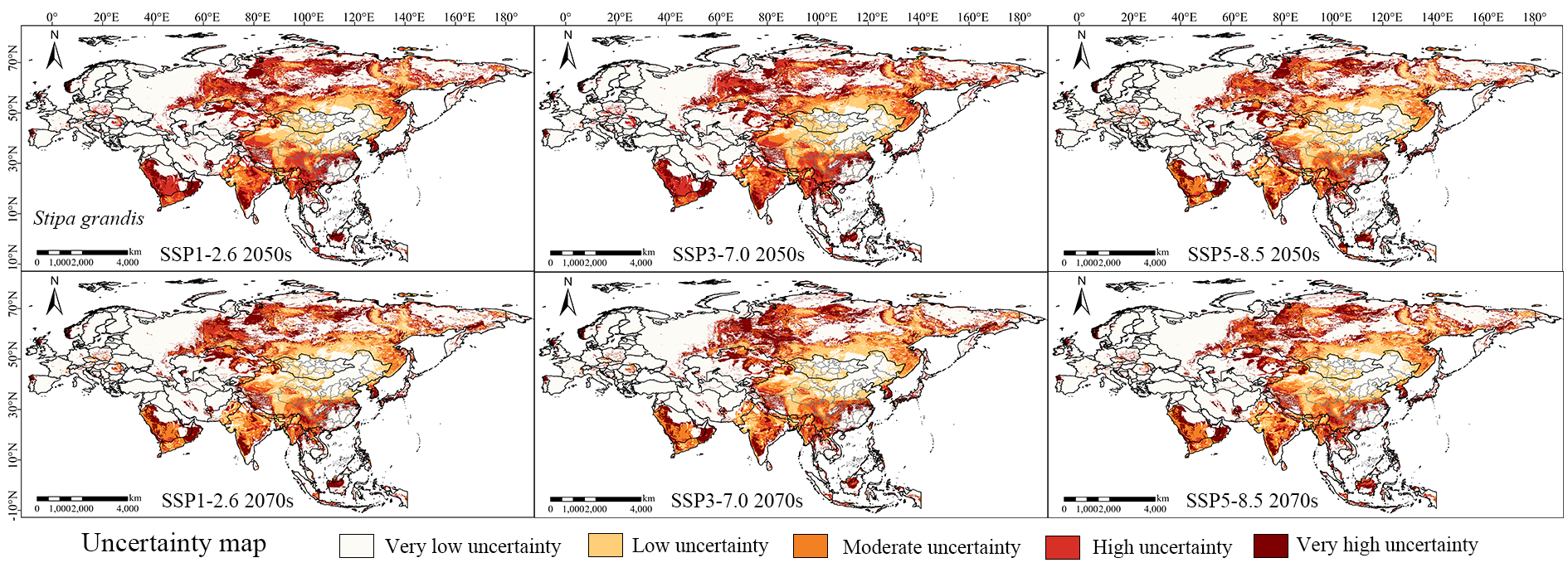


**Figure S15.** The uncertainty map of *Stipa grandis* in different time periods and different scenarios.


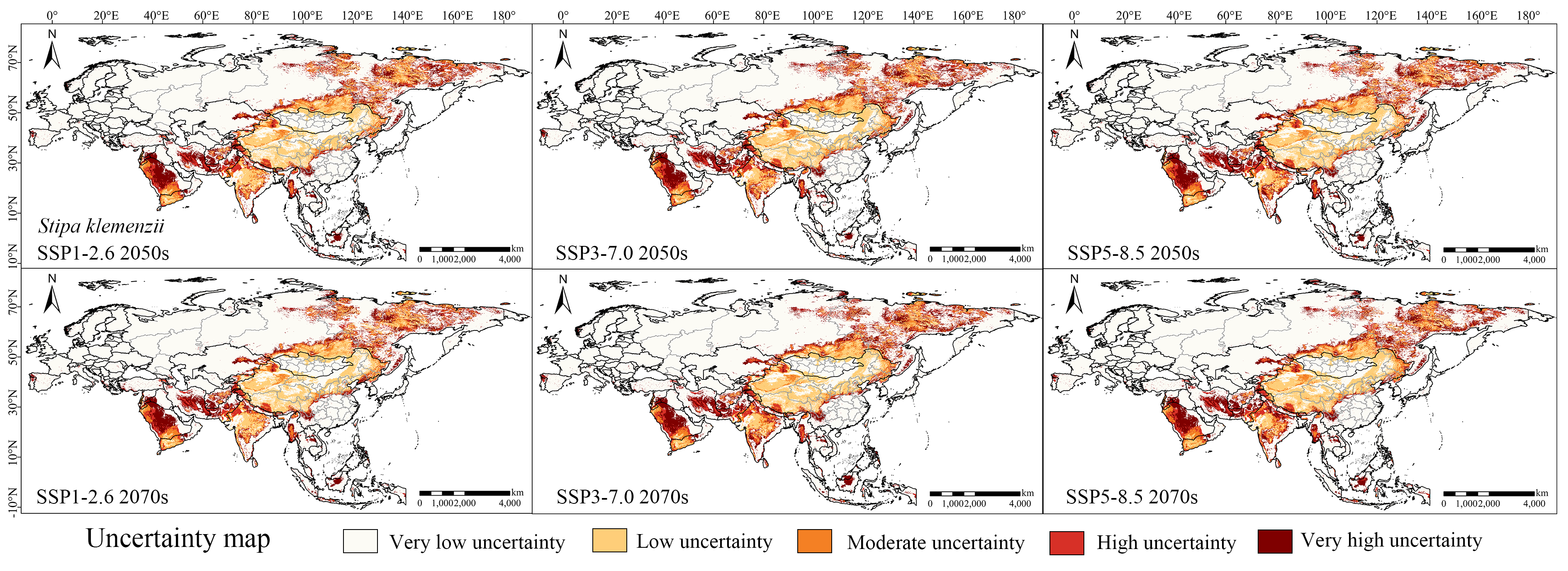


**Figure S16.** The uncertainty map of *Stipa klemenzii* in different time periods and different scenarios.

## Supplementary Tables

Table S1. Species distribution data sources.

| Full Name | *S. breviflora* | *S. bungeana* | *S. grandis* | *S. klemenzii* |
| --- | --- | --- | --- | --- |
| Specimens | 84 | 22 | 47 | 38 |
| GBIF | 24 | 31 | 64 | 9 |
| CVH, PE | 130 | 391 | 116 | 10 |
| *Flora of Siberia* | 0 | 0 | 6 | 3 |
| Literature | 0 | 0 | 86 | 29 |
| Total | 238 | 444 | 319 | 89 |

**Note:** GBIF source - Global Biodiversity Information Facility, CVH source - Chinese Virtual Herbarium, PE source - Chinese Academy of Sciences.

**Table S2**. Descriptions of the CMIP6 future climate scenarios (SSP) used in this study.

| Scenario | Scenario Name | 2100 radiation forcing |
| --- | --- | --- |
| SSP1-2.6 | Sustainability | 2.6 W/m^2^ |
| SSP3-7.0 | Regional Rivalry | 7.0 W/m^2^ |
| SSP5-8.5 | Fossil-fueled Development | 8.5 W/m2 |

Note: The first digit following "SSP" represents the specific Shared Socioeconomic Pathway, while the second digit denotes the total radiative forcing level (W/m2) by the year 2100.

**Table S3**. Environmental variables for four species

| Variable Type | Code(Unit) | Description | Stipa breviflora | Stipa bungeana | Stipa grandis | Stipa klemenzii |
| --- | --- | --- | --- | --- | --- | --- |
| Climatic  variables | Bio1 (℃) | Annual Mean Temperature | √ |  |  |  |
|  | Bio2 (℃) | Mean Diurnal Range | √ |  |  |  |
|  | Bio3 | Isothermality |  | √ |  |  |
|  | Bio4 | Temperature Seasonality |  |  | √ | √ |
|  | Bio5 (℃) | Max Temperature of Warmest Month |  |  |  |  |
|  | Bio6 (℃) | Min Temperature of Coldest Month |  |  |  |  |
|  | Bio7 (℃) | Temperature Annual Range |  |  |  |  |
|  | Bio8 (℃) | Mean Temperature of Wettest Quarter |  |  |  |  |
|  | Bio9 (℃) | Mean Temperature of Driest Quarter | √ |  |  | √ |
|  | Bio10 (℃) | Mean Temperature of Warmest Quarter |  |  |  |  |
|  | Bio11 (℃) | Mean Temperature of Coldest Quarter | √ | √ |  |  |
|  | Bio12 (mm) | Annual Precipitation |  |  |  |  |
|  | Bio13 (mm) | Precipitation of Wettest Month |  |  |  |  |
|  | Bio14 (mm) | Precipitation of Driest Month |  |  |  |  |
|  | Bio15 | Precipitation Seasonality |  | √ |  | √ |
|  | Bio16 (mm) | Precipitation of Wettest Quarter |  |  |  |  |
|  | Bio17 (mm) | Precipitation of Driest Quarter |  |  |  |  |
|  | Bio18 (mm) | Precipitation of Warmest Quarter | √ | √ | √ | √ |
|  | Bio19 (mm) | Precipitation of Coldest Quarter | √ | √ | √ | √ |
|  | Wind (ms-1) | Wind-mean |  |  | √ |  |
|  | Srad (KJ-2day-1) | Srad-mean | √ | √ |  |  |
|  | Vapr | Vapr-mean |  | √ |  |  |
| Topography | Elev (m) | Elevation | √ |  | √ | √ |
|  | Slop (°) | Slope | √ | √ |  |  |
|  | Aspect (°) | Aspect |  |  |  | √ |
| Soil Variables | Coarse (%) | D1- Coarse Fragments |  |  |  | √ |
|  | Sand (%) | D1- Sand Content |  |  |  |  |
|  | Silt (%) | D1- Silt Content |  |  |  |  |
|  | Clay (%) | D1- Clay Content |  |  |  |  |
|  | Texture | D1- Texture USDA |  |  |  |  |
|  | Bulk (g/cm3) | D1- Bulk Density | √ |  |  |  |
|  | Ref_bulk(g/cm3) | D1- Reference Bulk Density |  |  |  |  |
|  | Org_carbon (%) | D1- Organic Carbon Content |  |  |  |  |
|  | Ph_water (-log[H⁺]) | D1-pH in Water | √ |  |  |  |
|  | Total_N (g/kg) | D1- Total Nitrogen Content |  |  |  |  |
|  | CN_ratio (c/n) | D1-C/N Ratio |  |  |  |  |
|  | Cec_soil (cmol/kg) | D1-Cation Exchange Capacity |  |  |  |  |
|  | Cec_clay (cmol/kg) | D1-CECclay |  |  |  |  |
|  | Cec_eff (cmol/kg) | D1-Effective CEC |  |  |  |  |
|  | Teb (cmol/kg) | D1-Total Exchangeable Bases |  |  |  |  |
|  | Bsat (%) | D1-Base Saturation as Percentage of CECsoil | √ | √ | √ | √ |
|  | Alum_sat (%) | D1-Aluminum Saturation as Percentage of ECEC |  | √ | √ | √ |
|  | Esp (%) | D1-Exchangeable Sodium percentage |  |  |  |  |
|  | Tcarbon-eq (%) | D1-Calcium Carbonate Content | √ | √ |  |  |
|  | Gypsum (%) | D1-Gypsum Content | √ | √ |  | √ |
|  | Elec_cond (ds/m) | D1-Electric Conductivity |  |  |  |  |

**Table S4.** Model algorithms and default parameter settings.

| Algorithm | Abbreviation | Parameter settings |
| --- | --- | --- |
| Artificial Neural Network | ANN | size = 5; decay = 0.1; trace = FALSE; rang = 0.1; maxit = 200 |
| Classification Tree Analysis | CTA | xval = 5; minbucket = 5; minsplit = 5; cp = 0.001; maxdepth = 10 |
| Flexible Discriminant Analysis | FDA | method = "mars" |
| Generalized Additive Model | GAM | method = "GCV.Cp"; epsilon = 1e−06; trace = FALSE; maxit = 100 |
| Generalized Boosting Model | GBM | n.trees = 2500; interaction.depth = 7; n.minobsinnode = 5; shrinkage = 0.001; cv.folds = 3; keep.data = FALSE; n.cores = 1 |
| Generalized Linear Model | GLM | mustart = 0.5; maxit = 50 |
| Multivariate Adaptive Regression Splines | MARS | ncross = 0; penalty = 2; thresh = 0.001; pmethod = "backward" |
| Maximum Entropy | MAXENT | Implemented using maxent.jar with default settings |
| Random Forests | RF | ntree = 500; mtry = 2; nodesize = 5 |
| Random Forest Down-sampled | RFd | type = "classification"; ntree = 500; mtry = 2; strata = factor(c(0,1)); nodesize = 5 |
| Surface Range Envelope | SRE | do.extrem = TRUE |
| Extreme Gradient Boosting | XGBOOST | max_depth = 2; eta = 1; nthread = 2; nrounds = 4; objective = "binary:logistic" |

Note: All models were implemented in the R package biomod2 using customized parameter settings.

**Table S5**. Evaluation results of different models for four *Stipa* species

| species | model | AUC(mean+SD) | TSS(mean+SD) |
| --- | --- | --- | --- |
| *S. breviflora* | CTA | 0.915 ± 0.027 | 0.818 ± 0.061 |
|  | FDA | 0.946 ± 0.010 | 0.822 ± 0.039 |
|  | GAM | 0.949 ± 0.011 | 0.819 ± 0.42 |
|  | GBM | 0.973 ± 0.010 | 0.840 ± 0.036 |
|  | GLM | 0.970 ± 0.010 | 0.847 ± 0.040 |
|  | MARS | 0.970 ± 0.009 | 0.857 ± 0.047 |
|  | MAXENT | 0.976 ± 0.009 | 0.860 ± 0.048 |
|  | EM | 0.991 | 0.915 |
| *S. bungeana* | CTA | 0.937 ± 0.017 | 0.872 ± 0.028 |
|  | GAM | 0.952 ± 0.010 | 0.808 ± 0.035 |
|  | GBM | 0.981 ± 0.006 | 0.897 ± 0.032 |
|  | GLM | 0.981 ± 0.005 | 0.900 ± 0.025 |
|  | MARS | 0.982 ± 0.005 | 0.905 ± 0.027 |
|  | MAXENT | 0.986 ± 0.004 | 0.911 ± 0.025 |
|  | RF | 0.983 ± 0.006 | 0.867 ± 0.052 |
|  | RFd | 0.983 ± 0.006 | 0.893 ± 0.040 |
|  | XGBOOST | 0.963 ± 0.016 | 0.867 ± 0.035 |
|  | EM | 0.995 | 0.951 |
| *S. grandis* | CTA | 0.931 ± 0.022 | 0.844 ± 0.036 |
|  | GBM | 0.980 ± 0.008 | 0.878 ± 0.030 |
|  | GLM | 0.974 ± 0.014 | 0.876 ± 0.025 |
|  | MARS | 0.976 ± 0.010 | 0.876 ± 0.034 |
|  | MAXENT | 0.979 ± 0.008 | 0.879 ± 0.030 |
|  | RF | 0.979 ± 0.009 | 0.848 ± 0.040 |
|  | RFd | 0.979 ± 0.010 | 0.876 ± 0.039 |
|  | XGBOOST | 0.963 ± 0.020 | 0.856 ± 0.047 |
|  | EM | 0.99 | 0.934 |
| *S. klemenzii* | CTA | 0.886 ± 0.036 | 0.823 ± 0.044 |
|  | FDA | 0.939 ± 0.010 | 0.809 ± 0.058 |
|  | GAM | 0.948 ± 0.013 | 0.801 ± 0.067 |
|  | GBM | 0.970 ± 0.009 | 0.842 ± 0.055 |
|  | GLM | 0.964 ± 0.012 | 0.850 ± 0.059 |
|  | MARS | 0.963 ± 0.014 | 0.884 ± 0.031 |
|  | MAXENT | 0.973 ± 0.009 | 0.850 ± 0.037 |
|  | RFd | 0.976 ± 0.009 | 0.806 ± 0.064 |
|  | EM | 0.927 | 0.984 |

**Table S6.** Centroid sites of four species at different times.

| Species | Scenarios | Time | Centroid Point |
| --- | --- | --- | --- |
| *S. breviflora* | current | current | 97°56′31″E  39°38′4″N |
|  | SSP1-2.6 | 2050s | 97°21′3″E  39°33′35″N |
|  |  | 2070s | 97°9′28″E  39°28′44″N |
|  | SSP3-7.0 | 2050s | 96°42′6″E  39°20′4″N |
|  |  | 2070s | 95°49′44″E  39°18′13″N |
|  | SSP5-8.5 | 2050s | 96°31′E  39°25′9″N |
|  |  | 2070s | 95°30′11″  39°7′53″N |
| *S. bungeana* | current | current | 105°21′28″E  37°0′23″N |
|  | SSP1-2.6 | 2050s | 104°21′58″E  37°59′13″N |
|  |  | 2070s | 104°1′16″E  37°56′34″N |
|  | SSP3-7.0 | 2050s | 103°26′43″E  37°50′23″N |
|  |  | 2070s | 103°12′14″E  39°0′20″N |
|  | SSP5-8.5 | 2050s | 103°30′6″E  38°26′52″N |
|  |  | 2070s | 102°41′12″E  38°39′18″N |
| *S. grandis* | current | current | 112°4′54″E  46°25′26″N |
|  | SSP1-2.6 | 2050s | 110°51′29″E  45°51′N |
|  |  | 2070s | 110°44′14″E  45°47′12″N |
|  | SSP3-7.0 | 2050s | 110°39′6″E  45°48′6″N |
|  |  | 2070s | 110°45′35″E  45°47′31″N |
|  | SSP5-8.5 | 2050s | 110°33′12″E  45°56′17″N |
|  |  | 2070s | 110°40′14″E  45°42′49″N |
| *S. klemenzii* | current | current | 103°20′11.95″E  44°26′10.62″N |
|  | SSP1-2.6 | 2050s | 102°4′0.46″E  44°18′56.41″N |
|  |  | 2070s | 101° 39' 11.00" E  44° 11' 29.59" N |
|  | SSP3-7.0 | 2050s | 101°42′40.74″E  44°23′50.95″N |
|  |  | 2070s | 102°21′30.06″E  44°49′17.02″N |
|  | SSP5-8.5 | 2050s | 101°41′45.80″E  44°25′1.61″N |
|  |  | 2070s | 102°18′49.05″E  44°54′51.60″N |

**Table S7**. The centroid migration distance in different scenarios in the future.

| Species | Scenarios | Time period | Distance (km) |
| --- | --- | --- | --- |
| *S. breviflora* | SSP1-2.6 | current-2050s | 53.56 |
|  |  | 2050s-2070s | 19.33 |
|  | SSP3-7.0 | current-2050s | 115.84 |
|  |  | 2050s-2070s | 78.63 |
|  | SSP5-8.5 | current-2050s | 129.90 |
|  |  | 2050s-2070s | 96.30 |
| *S. bungeana* | SSP1-2.6 | current-2050s | 138.61 |
|  |  | 2050s-2070s | 32.14 |
|  | SSP3-7.0 | current-2050s | 198.54 |
|  |  | 2050s-2070s | 125.51 |
|  | SSP5-8.5 | current-2050s | 229.63 |
|  |  | 2050s-2070s | 77.57 |
| *S. grandis* | SSP1-2.6 | current-2050s | 115.03 |
|  |  | 2050s-2070s | 11.81 |
|  | SSP3-7.0 | current-2050s | 131.68 |
|  |  | 2050s-2070s | 8.65 |
|  | SSP5-8.5 | current-2050s | 131.63 |
|  |  | 2050s-2070s | 26.11 |
| *S. klemenzii* | SSP1-2.6 | current-2050s | 104.73 |
|  |  | 2050s-2070s | 36.50 |
|  | SSP3-7.0 | current-2050s | 132.96 |
|  |  | 2050s-2070s | 69.89 |
|  | SSP5-8.5 | current-2050s | 134.13 |
|  |  | 2050s-2070s | 73.66 |
